# Why probiotics fail: gut niche tension as the missing variable?

**DOI:** 10.1080/19490976.2026.2728333

**Published:** 2026-09-10

**Authors:** Nadezhda A. Pechnikova, Olga Zoikidou, Anita R. Denisova, Theodora Choli-Papadopoulou, Amalia Aggeli, Alexey V. Yaremenko

**Affiliations:** a Laboratory of Chemical Engineering A′, Department of Chemical Engineering, Faculty of Engineering, Aristotle University of Thessaloniki, Thessaloniki, Greece; b Saint Petersburg Pasteur Institute, Saint Petersburg, Russia; c Elpida BioPharm P.C., Thessaloniki, Greece; d School of Medicine, Aristotle University of Thessaloniki, Thessaloniki, Greece; e Department of Childhood Diseases of the Clinical Institute of Child Health named after N.F. Filatov, Sechenov First Moscow State Medical University (Sechenov University), Moscow, Russia; f School of Chemistry, Aristotle University of Thessaloniki, Thessaloniki, Greece

**Keywords:** Gut, microbial niche, probiotic, niche tension

## Abstract

Probiotic and live biotherapeutic interventions frequently yield inconsistent clinical benefits despite strong mechanistic rationale and widespread use. We propose that this heterogeneity reflects the ecological state of the gut at the time of dosing, in which an introduced strain must invade an already occupied ecosystem shaped by host physicochemical filters and resident community interactions. We introduce microbial niche tension as a trial-operational state variable capturing the net impedance to engraftment imposed by exclusionary forces (resource competition, antagonism, spatial crowding, and host immune and chemical barriers) and facilitative forces (niche vacancy, cross-feeding, and transient permissive windows). We further define a niche tension index (NTI) as a quantitative framework to estimate engraftment permissiveness across space, time, and host contexts. We synthesize evidence showing that niche tension is structured by gut transit, pH, oxygen gradients, diet, antibiotics, and host factors, including sex and hormonal state. Finally, we outline design rules for precision probiotics, including niche preconditioning, cooperative guild construction, sex-aware stratification, and data-driven prediction tools, to improve reproducibility and clinical efficacy.

## Graphical abstract

**Figure:**
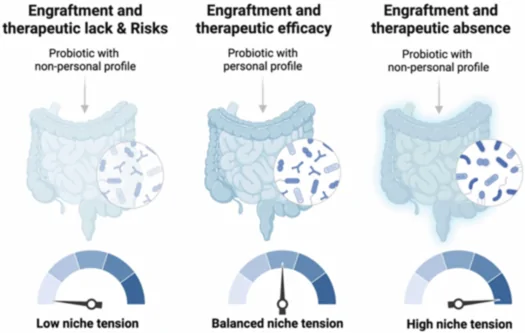

## Introduction

**Figure 1.**
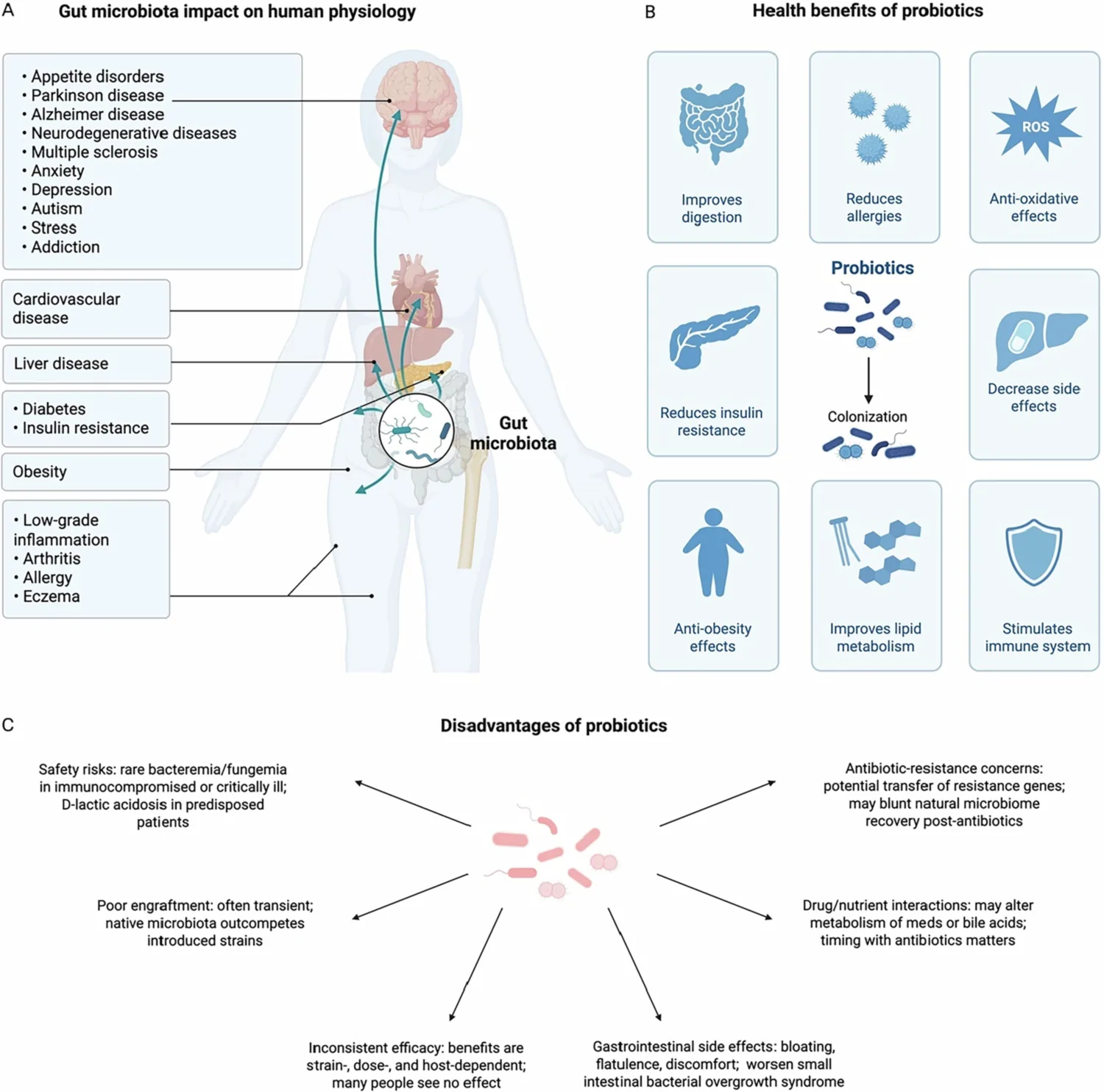
Gut microbiota–host interactions and the benefits and limitations of probiotic interventions. (a) Schematic overview of how the gut microbiota interfaces with multiple organ systems and disease domains, including neuropsychiatric/neurologic outcomes. The diagram illustrates bidirectional gut–organ communication (notably the gut–brain axis) with the gut microbiota as a central regulator. (b) Summary of the putative health benefits of probiotics, highlighting improved digestion, attenuated allergies, antioxidant effects, reduced insulin resistance, transient colonization/interaction with the gut ecosystem, decreased adverse effects, anti-obesity effects, improved lipid metabolism, and immune-system stimulation. (c) Key disadvantages/constraints of probiotics, including rare but relevant safety risks.

The human gut microbiota (GM) plays a vital role in maintaining health by forming axes with various organs, such as the brain, liver, and lung, etc.^1‐5^ Keeping them healthy and functioning properly involves GM's ability to ferment nutrients, produce essential vitamins, and help train the immune system. Knowledge of the role of the GM has led to the possibility of manipulating the gut ecosystem to improve the treatment outcomes of various diseases and health` conditions such as cancer,^6^ diabetes,^7^ obesity,^8^ depression,^9^ ^,^ ^10^ polycystic ovary syndrome,^11^ heart failure,^2^ stress responsiveness in a circadian manner (Figure 1A).^12^ One of the most prominent areas of microbiota manipulation is probiotics, which have been used for thousands of years, but were officially named only in 1965.^13‐15^ Probiotics, defined as “*live microorganisms which, when administered in adequate amounts, confer a health benefit on the host*”,^16^ are widely marketed and consumed for purported benefits ranging from digestive health to reducing mortality in preterm infants,^17^ preventing acute diarrhea^18^ and improving cancer therapy, etc (Figure 1B).^19^ However, the overall track record of probiotics in controlled trials has been paradoxical (Figure 1C), with several studies showing no full efficacy, minimal or no efficacy in otherwise promising indications.^20‐22^ New clinical guidelines reflect this discrepancy – the American Gastroenterological Association recently recommended against routine probiotic use for disorders like ulcerative colitis, Crohn's disease, or irritable bowel syndrome outside of clinical trials, citing insufficient high-quality evidence and inconsistent outcomes.^20^ Thus, the promise of probiotics, while compelling, is thus far only partially fulfilled, and this gap between expectation and reality poses a fundamental question: why do probiotics help in some cases but fail in others?

A critical piece of this puzzle could lie in the dynamic ecology of the gut. When a probiotic is taken, there are effectively introduced new species into an already occupied ecosystem, which is a vibrant community that can resist invaders -a phenomenon known as colonization resistance.^23‐25^

A healthy, balanced microbiome is adept at excluding newcomers—an evolutionary advantage for the host because it prevents pathogen colonization,^23^ but this is also a complicating factor for probiotic interventions. Antibiotic perturbation illustrates this vividly: after antibiotics disrupt the native flora and reduce diversity, colonization resistance is weakened, and probiotics (or opportunists) can more easily take hold.^26^ Conversely, in an unperturbed, high-diversity gut, even purportedly beneficial strains are often simply unable to find an open ecological niche to inhabit long-term. In addition, often-overlooked factor in probiotic engraftment is the role of sex,^27^ living together members in one family, the consumption of different drugs, including probiotics by members of one family, and having pets, which can also change the personal GM.^28^ Men and women differ in numerous physiological and immunological parameters, and emerging evidence suggests that these differences extend to the gut microbiome's composition and dynamics.^27^ The gut bacteria can metabolize sex hormones and modulate their availability,^27^ suggesting a bidirectional crosstalk between the endocrine system and microbiota. In addition, new insights into changes in the GM during gender transition are emerging.^29^ ^,^ ^30^ These differences may mean that the ecological landscape of the female gut is not identical to that of the male gut, affecting how probiotic organisms compete or cooperate within each. Yet most probiotic trials and studies to date have not stratified results by sex or examined sex-specific outcomes.^27^ A sex-aware ecology of probiotic engraftment is therefore needed, recognizing that factors such as hormones, immune responses, and even typical diets or medication exposures (which can differ between men and women) might modulate the success and effects of a given probiotic.

Also, high microbial diversity and functional redundancy in the gut mean that most nutritional and spatial niches are already filled; incoming bacteria often find no vacant niche and are outcompeted for nutrients and attachment sites.^23^ In the context of probiotics, this same effect can translate to poor engraftment—probiotic organisms survive transit and reach the gut, but then largely fail to establish a foothold because incumbent microbes and host factors create a hostile, competitive environment. Also, the ability of a given probiotic strain to stably engraft appears to depend on individualized features of the host and host's microbiome.^26^ For example, a controlled trial of a probiotic cocktail in adults with metabolic syndrome found that only a subset of individuals—those with certain pre-existing microbiome and diet profiles derived metabolic benefits, whereas others (with different gut ecology) saw no improvement or even adverse shifts in biomarkers.^22^ These results emphasize that the use of probiotics requires a more detailed analysis of the patient's profile and gut profile for its survival or functioning. Moreover, the gut environment itself imposes stringent filters from the gauntlet of gastric acidity and bile salts to defensins and IgA in the intestine that most swallowed probiotics cannot survive. Those that do survive must then contend with the native microbiota's competitive tactics—competition for nutrients and attachment sites, production of antimicrobial compounds, and modulation of local pH and oxygen levels.^31‐33^ All these factors together create what we can term “microbial niche tension” in the gut. This term is not in position to establish concepts of resistance to colonization but complements it.

Colonization resistance is most often treated as an emergent outcome of resident microbiota and host barriers—i.e., the observed ability of the gut ecosystem to prevent the establishment of an incoming microbe.^34^ In contrast, ecological invasion theory describes a process from an arrival to a spread, governed by propagule pressure, abiotic filtering, and biotic interactions, but it is rarely written in a way that is immediately “trial-operational” for live biotherapeutics.^33^

This framing is consistent with foundational microbial invasion ecology. Experimental work in soil microcosms has shown that resident microbial diversity can determine invasion resistance to an introduced bacterial pathogen, supporting the idea that filled niches and functional redundancy constrain establishment. Conceptual and review-level microbial invasion frameworks further define invasion as a staged process involving introduction, establishment, growth, spread and impact, governed by dispersal or propagule pressure, selection by environmental filters, ecological drift, diversification and resident-community properties.^33^ ^,^ ^35^ ^,^ ^36^

Human probiotic studies further show that success is highly context-dependent, with “personalized” mucosal colonization resistance and variable post-antibiotic recovery trajectories across individuals.^25^ Building on these foundations, we introduce microbial niche tension as a state variable—the net ecological impedance encountered by an introduced strain at a specific time and gut location-integrating (i) exclusionary forces (competition, antagonism, host physicochemical/immune filters) and (ii) facilitative forces (niche vacancy, cross-feeding, supportive diet/perturbation windows) that together determine whether engraftment is permissive or resistant, consistent with ecological frameworks for engraftment and human strain-level engraftment determinants after microbiome interventions.^32^ Thus, niche tension refers to the multifaceted ecological pushback that a probiotic strain encounters as it tries to occupy a niche in the human gut. It encapsulates the idea of a constant tug-of-war between the introduced microbe's drive to engraft and the array of forces (resident microbes plus host-mediated factors) that oppose it.

In light of these ideas, this review aims to examine in detail when, where and for whom probiotics are likely to be effective through the lens of niche tension. By taking a comprehensive, personal, sex-specific ecological approach, we aim to provide a roadmap to make probiotic interventions more predictable, personalized and effective in the coming years.

Despite compelling mechanistic rationale and widespread consumption, probiotics and live biotherapeutic products show highly variable and often disappointing efficacy in controlled human trials.

## Mapping niche tension in the human gut

### Microbial niche tension as a determinant of colonization resistance and engraftment

Microbial niche tension is the dynamic ecological balance of competitive and cooperative interactions among microorganisms within human-associated microbiomes, where limited space, nutrients, and host-derived factors drive conflict (e.g., competitive exclusion, metabolic antagonism) and coexistence (e.g., mutualism, cross-feeding) to shape community structure.

**Figure 2.**
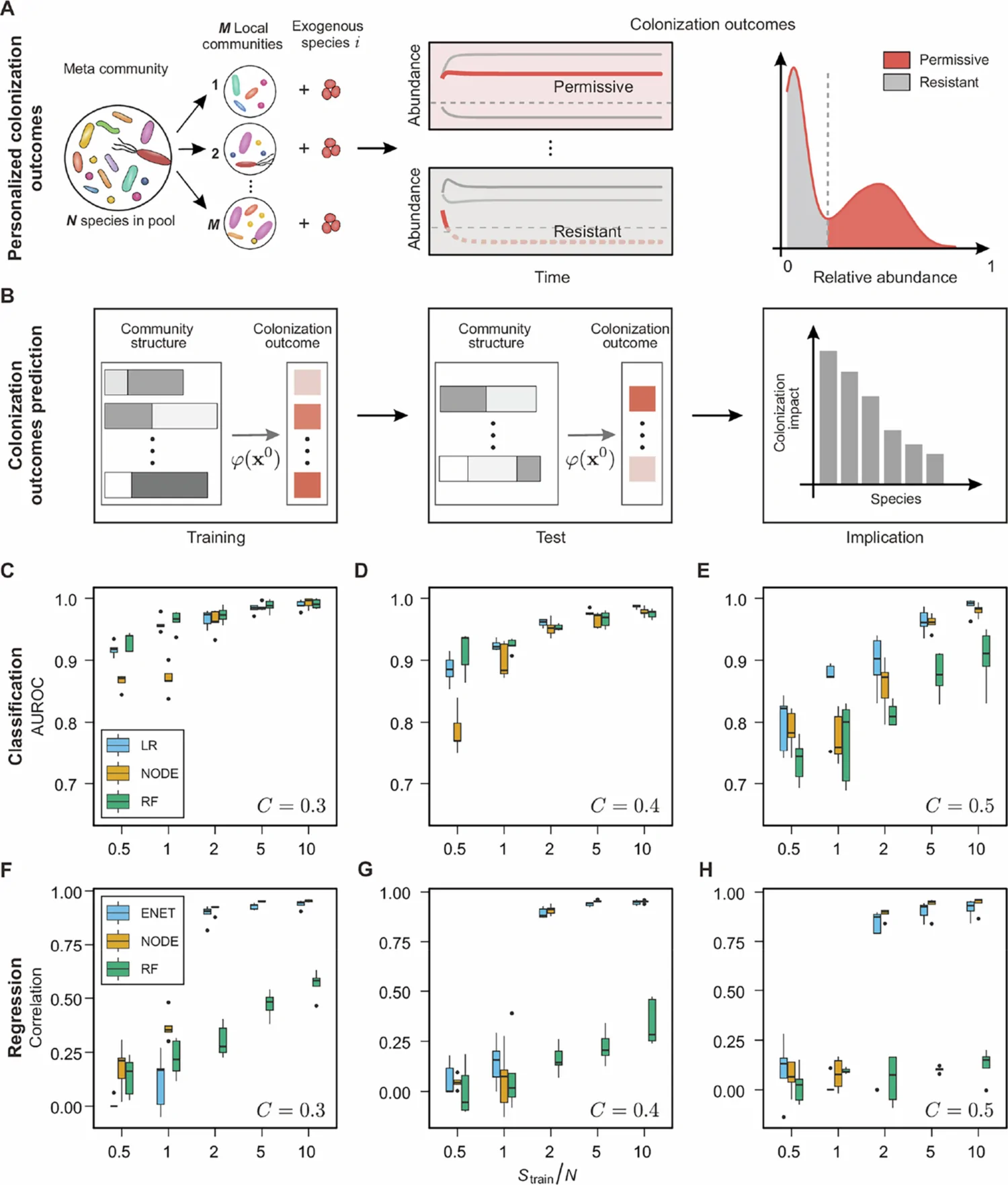
Predicting colonization outcomes in complex microbial communities using a data-driven framework.^26^ (a) Each person's microbiome can be conceptualized as a local ecological community, representing a subset of the broader metacommunity of microbial species. When an exogenous species invades these local communities, its colonization outcome (e.g., permissive & resistant) can be highly individualized, depending on the composition of the local community. (b) Colonization outcome prediction (COP) can be addressed by learning a mapping from the baseline taxonomic profile to the post-invasion abundance of the exogenous species (i.e., its abundance after invasion/intervention). (c–e) Evaluation of the data-driven framework for COP as a classification task. Performance is reported as AUROC for three machine-learning models: logistic regression (LR), a COP–neural ordinary differential equations classifier (COP-NODE; NODE), and a random forest classifier (RF). (f–h) Evaluation of the data-driven framework for COP as a regression task. Performance is reported as the Pearson correlation between true abundance and model-predicted abundance for three models: elastic net linear regression (ENET), a COP-NODE regressor (NODE), and a random forest regressor (RF), tested under network connectivity C = 0.3, 0.4, 0.5. The error bars indicate 95% confidence intervals; box plots show the 25th–75th percentiles (box), 50th percentile (median line), and outliers as individual points. Reproduced under terms of the CC BY-NC-ND license.^26^ 2024, Wu et al., published by Springer Nature.

This tension underlies the gut's colonization resistance -the ability of resident microbiota to fend off invaders by consuming shared resources, producing inhibitory compounds, and priming host defenses.^26^ A diverse, well-filled community tends to occupy available niches with functional redundancy, leaving little room for newcomers.^26^ Indeed, data-driven models show that a microbiome's starting composition can predict whether an incoming species will establish and at what abundance (Figure 2A–H).^26^

Consistently, experiments find that single probiotic strains usually offer minimal protection, whereas a consortium of many commensals can suppress pathogens by several orders of magnitude.^23^ Such findings point to a measurable “tension index” of competitive capacity, driven by factors like (i) species diversity/species richness, (ii) functional redundancy, and (iii) resource overlap with potential invaders – parameters that reflect how tightly the niche is filled and how forcefully incumbents can resist ecological newcomers. These parameters mirror the core determinants of microbial invasibility described in earlier invasion ecology studies: resident-community diversity and evenness, resource availability, invader supply and environmental filtering. In this sense, NTI does not replace colonization resistance or invasion ecology but converts their central determinants into trial-measurable variables that can be estimated before dosing.^33^ ^,^ ^35^ ^,^ ^37^

To make the concept measurable, we define the NTI as the normalized balance between exclusionary forces *E* and facilitative forces *F* acting on a candidate strain in a given host:

$$\mathrm{NTI}=\frac{E-F}{E+F+\varepsilon }\in [-1,1],$$

where is a small positive constant is added to the denominator to prevent division by zero (or unstable values) when *E + F* is very small, NTI + 1 indicates a high-tension, invasion-resistant niche; NTI ≈ 0 indicates a contested/neutral niche; and NTI −1 indicates a permissive, low-tension niche. In practice, *E* can be parameterized using trial-measurable proxies such as community occupancy/diversity, functional redundancy, resource overlap, and host filtering metabolites (e.g., bile-acid landscape), which are all empirically linked to resilience/engraftment barriers and colonization resistance.^38^ Conversely, *F* captures niche vacancy and facilitation (including cross-feeding and “priority effects,” where early colonizers can pre-empt or modify niches to inhibit-or sometimes facilitate-later arrivals), supporting a quantitative bridge from ecological assembly theory to responder stratification and niche preconditioning in live biotherapeutic product/probiotic trials.^39^ NTI can be connected to colonization outcome as a probabilistic forecast with parameters estimated from trial data where engraftment is measured (strain-resolved metagenomics/qPCR):

$$P(\text{engraft})=\frac{1}{1+\exp(\alpha \cdot \mathrm{NTI}+\beta )},$$

where $e^{x}$ is the Euler's number e≈2.718 , α is a fitted slope/scaling parameter that determines how strongly NTI changes the probability (with α>0 , higher NTI lowers P ), and β is a fitted intercept that sets the baseline probability when NTI=0 . A minimal, trial-measurable proxy set for estimating the exclusionary *E* and facilitative *F* components of niche tension should capture community occupancy/competitive pressure, metabolic substitutability, resource landscape compatibility, and host filtering. Specifically, *E* can be approximated from baseline α-diversity and evenness as occupancy metrics, the abundance of nearest taxonomic and/or functional neighbors of the introduced strain as a proxy for niche pre-emption, and community-scale growth potential indicators inferred from metagenomic or compositional features.

Additional exclusionary pressure can be quantified via functional redundancy, operationalized as redundancy of metabolic pathways overlapping the predicted niche of s, and resource overlap, defined as the overlap between the predicted substrate utilization profile of s and the community's metabolic capacity. Host-derived constraints contributing to *E* should include the bile-acid landscape, stool pH, proxies of intestinal transit time, and inflammation markers that reflect immune and physicochemical filtering. Conversely, *F* can be estimated using indicators of niche vacancy (low abundance or absence of competing guilds), cross-feeding potential (availability of metabolites/substrates matching s's requirements), the availability of paired prebiotics or dietary substrates designed to support s, and perturbation timing, such as post-antibiotic windows that transiently increase permissiveness to engraftment.

**Figure 3.**
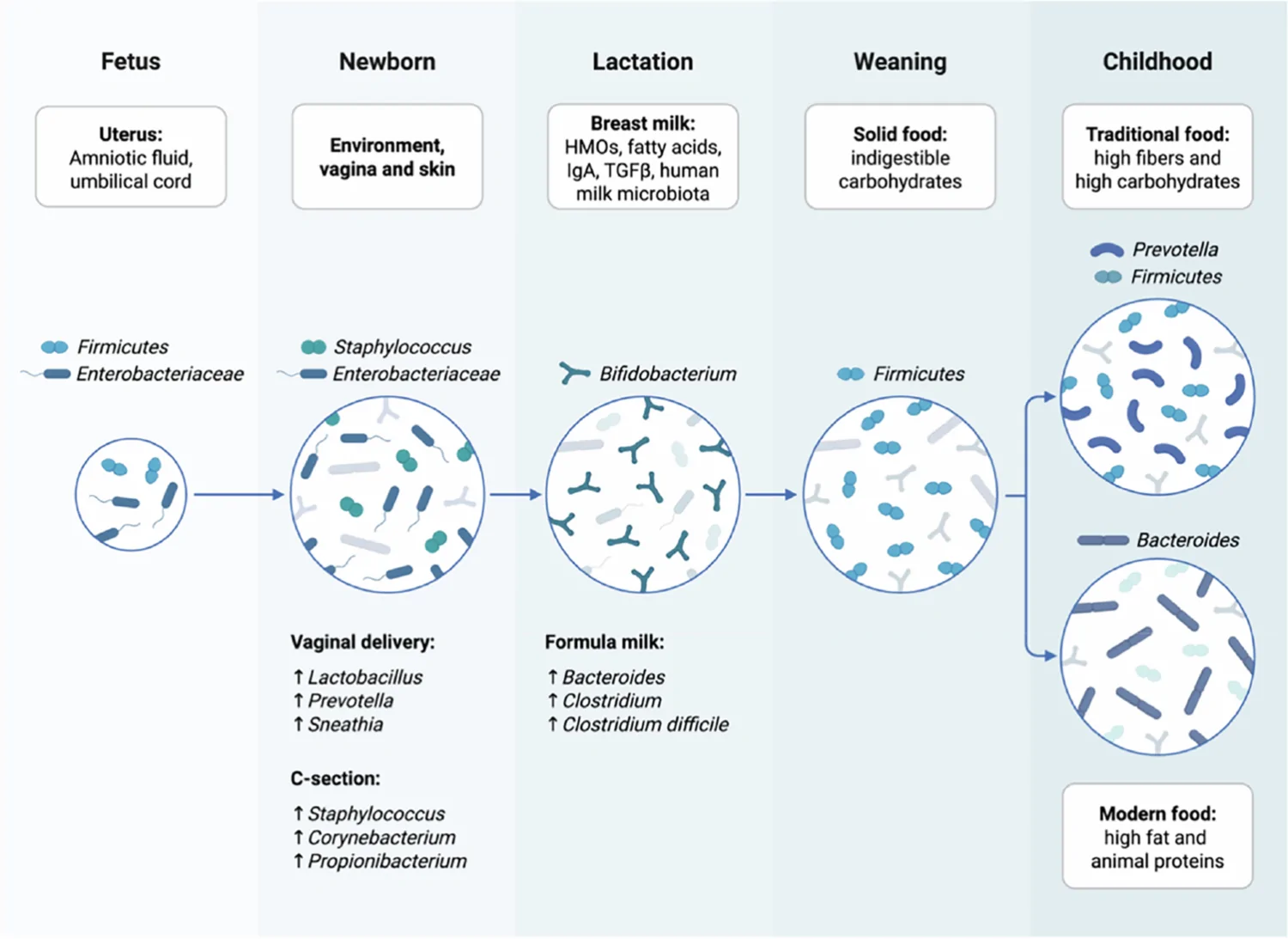
Microbiome development from fetus to childhood. This schematic illustrates the dynamic establishment and maturation of the human microbiome across early life stages, from the fetal period through childhood. During fetal development, microbial exposure is minimal, with the intrauterine environment largely shaped by maternal factors and limited microbial signals from the amniotic fluid and umbilical cord. At birth, initial microbial colonization is driven by environmental exposure, particularly from the vagina and skin, with delivery mode influencing early community structure. Vaginal delivery is associated with enrichment of *Lactobacillus* and *Prevotella*, whereas cesarean section favors skin- and environment-associated taxa such as *Staphylococcus*, *Corynebacterium*, and *Propionibacterium*. During lactation, breast milk–derived components-including human milk oligosaccharides (HMOs), fatty acids, immunoglobulins (IgA), TGF-β, and milk-associated microbes-selectively promote the expansion of *Bifidobacterium* and shape immune development. Formula feeding, in contrast, is associated with increased abundance of *Bacteroides* and *Clostridium* species, including *Clostridium difficile* (*C. difficile*). With weaning and the introduction of solid foods, the microbiome diversifies, showing increased representation of Firmicutes and enhanced metabolic capacity for complex carbohydrates. By childhood, dietary patterns become a dominant determinant of microbiome composition: traditional high-fiber diets favor *Prevotella* and *Firmicutes*, whereas modern diets rich in fat and animal protein promote *Bacteroides*.

Also, it should be noted that spatial organization further modulates niche tension. Gut microbes are not evenly distributed across gut sections; instead, distinct regions and microhabitats harbor unique communities. For instance, *in vivo* gut mapping has shown that a segment of the mid-colon, uniquely regulated by immune signals, creates a closed microbial community distinct from those above or below it.^40^ Moreover, high-resolution spatial sampling of human stool identified micron-scale “spatial hubs” – stable clusters of co-occurring bacteria (often dominated by *Bacteroidaceae*, *Ruminococcaceae*/*Lachnospiraceae* families) that recur across individuals.^41^ When the environment shifts (e.g., adding fermentable fiber inulin to the diet), these microscale communities reorganize and form new local partnerships, then revert once the perturbation ends.^41^ Temporal factors also shape niche tension. Early in life, the gut microbiome undergoes a predictable succession: pioneers such as *Bifidobacterium* (nourished by milk) dominate infancy and then yield to anaerobes (e.g., *Faecalibacterium*, *Lachnospiraceae*) as the diet diversifies (Figure 3).^42^

Disrupting this sequence (via cesarean section, antibiotics, *etc*.) can derail normal assembly, whereas seeding infants with keystone taxa (such as *Bifidobacterium* or *Bacteroides*) steers the community toward a balanced, resilient state.^42^ These priority effects—where early arrivals influence later ones – mean that timing is critical. For example, a suboptimal colonizer (*Bifidobacterium longum*) may initially occupy a niche, but a better-adapted species (*Bifidobacterium infantis*) introduced later can overtake it if the right nutrient (breast milk oligosaccharides) is present.^42^ Similarly, in adults, introducing a new beneficial strain often succeeds only if an ecological window is open (such as after antibiotic clearance); otherwise, the entrenched microbiota excludes the invader.^26^ Thus, niche tension in the human gut emerges from competitive exclusion and cooperative interplay among microbes, varying across space and time, and it fundamentally governs community stability and the host's resistance to perturbations.^26^

Importantly, recent microbiome-intervention studies indicate that engraftment should not be interpreted as a direct surrogate of clinical outcome. For example, it was found that although higher donor and recipient α-diversity and enrichment of butyrate-associated taxa were linked to better response during ulcerative colitis treatment, donor microbiota engraftment itself could not be clearly linked to clinical remission, suggesting that the ecological and functional quality of the transferred community may be more important than the donor take-rate alone.^43^ This caution is strengthened by other study showing that apparent post-FMT engraftment can be substantially overestimated unless placebo sequencing controls are included, indicating that some “engraftment” signals may reflect analytical noise rather than biologically meaningful colonization. Consistently, in the randomized phase 2 TACITO trial in metastatic renal cell carcinoma, the acquisition or loss of specific strains, but not total engraftment, was associated with the primary clinical endpoint, arguing that the outcome depends on which functions and taxa are transferred rather than on bulk engraftment alone.^44^ Likewise, the PERFORM trial linked durable engraftment of anti-inflammatory taxa and metabolic functions with reduced immune-related toxicity and improved response, whereas enrichment of donor-derived pro-inflammatory enzymes tracked with toxicity, again showing that engraftment can be beneficial, neutral, or harmful depending on its functional payload.^45^

Also, recent cancer study that have looked more closely at the effectiveness of fecal microbiota transplantation in combination with immunotherapy suggest that better results are achieved by eliminating the pathogenic microbe rather than by introducing new strains.^46^

Together, these data support the view that clinically meaningful engraftment is not mere donor detection in stool but durable, spatially relevant, strain- and function-specific niche occupancy.

### Niche tension versus colonization resistance and invasion ecology terms

In classical terms, colonization resistance is the emergent phenomenon whereby an established gut microbiota (and host barriers) prevents an incoming microorganism from taking hold.^34^ It is observed after the fact (did the invader succeed or fail?) and does not specify why it could be due to occupied niches, antimicrobial metabolites, or immune factors.^34^ In contrast, niche tension is defined here as a pre-engraftment ecological state: the balance at a specific gut site and time between factors blocking an invader (e.g., resource overlap, antagonistic metabolites, bile/oxygen/immune filtering) and factors enabling it (e.g., vacant niches, cross-feeding opportunities, or a recent antibiotic-induced “permissive window”). A practical complication is that many proposed proxies of niche tension are often measured after product exposure, when they may already reflect live biotherapeutic activity, failed colonization or niche remodeling rather than the true permissive state of the host ecosystem. This problem is particularly important because probiotic colonization in humans is highly individualized and depends on baseline host–microbiome features, and stool shedding does not necessarily capture mucosal colonization resistance.^25^ Therefore, niche tension should be defined operationally as a pre-exposure state variable measured before first contact with the live biotherapeutic product. Human trials should incorporate a baseline run-in phase with repeated pre-dose sampling, ideally including stool collected over several days before dosing, because the gut-community structure and metabolite profiles show substantial temporal variability and are shaped by stool consistency, fecal water content and intestinal transit time.^47^ ^,^ ^48^ Medication and diet metadata should be captured prospectively, as proton-pump inhibitors, laxatives, antibiotics and metformin are among the strongest drug-associated modifiers of the gut microbiome composition and function.^49‐51^ Baseline profiling should combine strain-resolved metagenomics with targeted metabolomics, bile-acid profiling, short-chain fatty acids (SCFA) quantification, fecal pH, water content and inflammatory markers such as calprotectin. When feasible, rectal swabs, mucosal biopsies or luminal aspirates should complement stool sampling because mucosal and fecal communities can differ, and mucosal sampling may better reflect colonization resistance at the target niche.^25^ For large probiotic or live-biotherapeutic trials, this does not mean that invasive sampling should be applied to every participant. A feasible alternative is a tiered sampling design in which each sampling layer answers a different question. The core sampling layer should include repeated stool collection in all participants because stool remains the most scalable matrix for baseline ecological screening, NTI estimation, longitudinal strain tracking and metabolomic profiling. A second, spatial-validation layer should be applied to a prespecified representative subset of participants using non-invasive ingestible sampling capsules, because intestinal microbial, proteomic and bile-acid profiles can differ substantially from stool and may reveal region-specific ecological states that are not visible in fecal samples.^52^ A third, mechanistic substudy layer should reserve rectal swabs, mucosal biopsies or luminal aspirates for responder/non-responder comparisons, high-priority validation cohorts or participants already undergoing clinically indicated endoscopy, which is consistent with evidence that stool shedding does not necessarily capture individualized mucosal colonization resistance to empiric probiotics.^25^ This design preserves feasibility for multicenter trials while testing whether stool-derived NTI estimates reflect the mucosal and segment-specific niches that determine probiotic engraftment.

These pre-dose variables should be modeled prospectively as predictors of later strain persistence, product-derived metabolites and clinical response, whereas post-dose changes in the same variables should be analyzed separately as mediators or consequences of engraftment. This temporal separation is supported by FMT studies showing that donor-strain engraftment varies across diseases, is influenced by recipient baseline ecology and antibiotic exposure, and is associated with clinical success.^53^ ^,^ ^54^ It is also supported by metabolomic studies showing that bile-acid and SCFA profiles shift after microbial transfer.^55^ ^,^ ^56^ The distinction is therefore essential to avoid circular inference, in which a post-engraftment ecological change is misclassified as a pre-engraftment determinant. In other words, colonization resistance is an outcome; niche tension is a quantifiable property one can be measured or estimated before dosing.

Because colonization resistance and microbial niche tension partially overlap, their distinction is clarified in Table 1. Invasion ecology offers the broader lens, defining microbial invasion as “the establishment of an alien microbial type in a resident community”.^36^ Foundational microbial invasion frameworks describe this process as a sequence of introduction, establishment, growth, spread and impact, shaped by dispersal or propagule pressure, abiotic filtering, biotic interactions, ecological drift and diversification.^36^ Experimental microbial invasion studies further show that resident diversity and community structure can determine invasion resistance, whereas propagule pressure can alter invasion success under some conditions. However, these frameworks are not by themselves patient-operational: they identify general ecological processes but do not specify which baseline host–microbiome variables should be measured before dosing in a probiotic or live-biotherapeutic trial.^33,35‐37^

**Table 1.** Distinction between colonization resistance and microbial niche tension.

Dimension	Colonization resistance	Microbial niche tension
Core definition	The ability of the resident gut microbiota, together with host barriers, to prevent an incoming microorganism from establishing in the gut^57^	A proposed pre-engraftment ecological state variable describing the net difficulty faced by an introduced probiotic/live biotherapeutic strain in occupying a specific gut niche
Conceptual status	Established concept in microbiome ecology and infection biology, mainly used to explain resistance to pathogen or exogenous-strain colonization^24^ ^,^ ^25^ ^,^ ^57^	New trial-operational framework proposed in this review to translate colonization resistance and invasion ecology into measurable variables for probiotic/LBP studies
Temporal scope	Usually inferred after exposure, based on whether the incoming organism colonized, persisted or failed to engraft^25^	Estimated before first product exposure, then followed longitudinally after dosing to distinguish pre-engraftment predictors from post-engraftment remodeling
Main question	Did the resident ecosystem prevent colonization?^57^	How resistant or permissive is this host niche for this strain at this time?
Ecological meaning	Describes the observed outcome of ecological exclusion	Describes the balance of forces that makes exclusion, transient passage or durable engraftment more likely
Main exclusionary forces	Nutrient competition, niche exclusion, antimicrobial metabolites, direct microbial antagonism, bile acids, epithelial barriers and immune-mediated host defence^58^	The same exclusionary forces, but treated as measurable components of an index: resident guild occupancy, α-diversity/evenness, functional redundancy, resource overlap, antagonism and host physicochemical filtering
Facilitative forces	Usually not central to the definition; the focus is mainly on barriers that prevent invasion^57^	Explicitly includes permissive factors such as niche vacancy, reduced resident competition, cross-feeding, compatible dietary substrates and post-antibiotic windows
Measurement approach	Commonly measured indirectly through post-intervention outcomes: pathogen suppression, probiotic persistence, strain abundance or engraftment failure^25^	Estimated from pre-treatment and longitudinal variables, including baseline microbiome composition, functional redundancy, resource overlap, bile-acid profile, stool pH, transit time, inflammation markers, diet and antibiotic exposure
Quantitative readout	Engraftment success/failure, abundance of the introduced strain, duration of persistence, or degree of pathogen suppression^25^	A niche tension index or related predictive score estimating the probability of strain engraftment or therapeutic function in each hos
Relationship to invasion ecology	Closely related to invasion failure, but usually does not formalize the full invasion sequence of arrival, establishment and spread^59^	Applies invasion ecology prospectively to clinical trial design by focusing on patient-specific barriers, facilitative windows and strain–niche compatibility
Implication for probiotic/LBP trial design	Explains retrospectively why an administered strain failed to colonize or why a pathogen was excluded)^25^	Can be used prospectively to stratify participants, select compatible strains, define dosing windows, add prebiotics/dietary substrates, or apply niche preconditioning
Intervention logic	Attempt to overcome resistance by increasing dose, repeating administration, using multi-strain consortia or selecting more competitive strains^32^	Reduce niche tension before or during dosing by lowering competition, exploiting antibiotic/dietary windows, providing exclusive nutrients, or matching the strain to an underoccupied niche [60]
Example interpretation	The probiotic failed because the resident microbiota resisted colonization^25^	The probiotic failed because the host had high niche tension: high occupancy of the target guild, strong resource overlap, no compatible substrate and unfavorable host filtering
Main limitation	Often retrospective and descriptive; it may identify that colonization failed without specifying which barrier was decisive​​​​​​^57^	Requires standardized proxy selection, weighting, normalization and validation before it can function as a robust clinical-trial tool

Niche tension explicitly applies these ideas to clinical trials: it highlights the patient-specific ecological variables that should guide design. For instance, rather than treating “resistance” as uniform, one might stratify or select participants based on baseline occupancy of the target niche (measured by the existing abundance of similar strains) and check substrate availability (dietary fibers, glycans) or recent disturbances (antibiotics, illness). These distinctions change the trial strategy. Dose escalation or adding strains will only help if a niche is incompletely occupied or if other barriers are low. Three recent trials illustrate how niche tension explains results better than traditional terms. In a 12-week RCT of pasteurized *Akkermansia muciniphila* (*A. muciniphila* strain AKK-WST01) in obese type-2 diabetics, the overall cohort had no significant difference & placebo.^61^ Crucially, in patients with low baseline *A. muciniphila*, the probiotic did colonize, and these patients saw significant reductions in body weight, fat mass and HbA1c; whereas in patients with high baseline *A. muciniphila*, the probiotic showed poor engraftment and no metabolic benefit^61^. Thus, the baseline occupancy of *Akkermansia*— not the dose—determined both engraftment success and clinical effect. In a 2-month trial of 120 gout patients on febuxostat, coadministration of a multi-strain probiotic (*Probio-X*) significantly reduced serum uric acid and gout attacks on average. However, post hoc analysis showed that only a “probiotic-responsive” subgroup achieved these improvements; the “non-responder” subgroup was indistinguishable from placebo. The investigators even built a metagenomic classifier showing that baseline gut microbiome composition predicted who would respond.^62^ In other words, patient-to-patient ecological differences—not probiotic compliance—determined engraftment and effect. Also, in the phase-3 NICU trial randomized preterm infants to a 28-d multispecies probiotic (including *B. infantis* BB-02) or placebo.^63^ The probiotic shifted the microbiota toward a more “eubiotic” profile, but it failed to reduce colonization by multidrug-resistant organisms (the primary endpoint). Strikingly, metagenomic analysis found the same *B. infantis* strain in 49% of placebo infants. Its presence was highly variable by site and strongly linked to having a treated sibling.^63^ This means environmental exposure and sibling transmission (niche availability in NICUs)—not trial arm assignment -drove engraftment of that strain. Traditional reports might call this a “breach” in trial protocols, but niche tension attributes it to the opening of ecological niches and high propagule pressure in certain hospital environments. In another 18-week RCT of a multi-strain probiotic in adults with metabolic syndrome, no overall benefit was seen. Instead, a subset of “responders” in the probiotic arm showed significant drops in triglycerides and blood pressure, while non-responders saw no benefit or even worsened glucose.^22^ Importantly, these responder differences aligned with diet: responders had distinct dietary patterns and gut microbiomes at baseline. This suggests that the probiotic required certain nutrient substrates or gut conditions (a facet of niche tension) to engraft and exert effects, which only some participants had.^22^

These cases share a common theme: the success or failure of a probiotic could not be explained by dose, strain, or compliance alone, but also by patient-specific ecology. Recognizing this, a niche tension framework would lead to trials that screen for or modulate those ecological factors: selecting subjects with low baseline occupancy, co-administering prebiotics or diets that fill required substrates, sampling multiple sites (e.g., using intestinal capsules) to verify engraftment, and using engraftment/readout endpoints.

### Analytical workflow for NTI standardization, weighting, and validation

For the NTI to move from a conceptual framework to a trial-operational tool, its calculation must follow a predefined and reproducible analytical workflow. This workflow should specify how raw ecological, functional and host-derived measurements are converted into standardized and directionally aligned exclusionary-force proxies *E* and facilitative-force proxies *F*, how these proxies are rescaled, weighted and combined, and how the resulting index is validated against observed engraftment or clinical-response outcomes.

Without such standardization, NTI estimates could be distorted by differences in measurement scale, sequencing depth, missing metadata, batch effects, compositional constraints, outliers, low-biomass contamination and cohort-specific technical noise.^64‐66^

The first step is to define the candidate proxy set before analysis. Exclusionary proxies should capture ecological barriers that make engraftment less likely, including high resident-community occupancy, high α-diversity and evenness, an abundance of close taxonomic or functional competitors of the introduced strain, metabolic-pathway redundancy, resource overlap, antimicrobial-gene burden, bile-acid-mediated filtering, unfavorable stool pH, rapid or delayed intestinal transit, inflammation and host immune activation. Facilitative proxies should capture factors that increase niche permissiveness, including a low abundance of competing guilds, reduced functional redundancy, niche vacancy after antibiotic exposure, the availability of compatible dietary substrates or prebiotics, cross-feeding potential, low antagonistic pressure and transient ecological windows created by perturbation or disease state. Each proxy should be assigned an expected directionality before modelling: for example, higher resource overlap with resident taxa is expected to increase *E*, whereas the availability of a substrate specifically required by the introduced strain is expected to increase *F*.

Before proxy integration, raw data require quality control. For sequencing-based variables, samples should be filtered using predefined thresholds for read depth, sequencing quality, host-DNA contamination, negative-control signal, batch completeness and sample-processing metadata. This step is especially important for low-biomass samples, because reagent contaminants and cross-contamination can dominate the microbial signal and generate false taxon–outcome associations.^65^ Negative controls, extraction blanks, mock communities and replicate samples should therefore be included wherever possible. Taxa or functions with extremely low prevalence should be removed or analyzed separately because sparse features can introduce instability, inflate multiple-testing burden and increase the risk of false-positive associations.

For metabolomic, bile-acid, cytokine, pH or transit-related variables, assay-specific quality control should be applied before transformation. Values below the limit of detection should be handled according to a predefined rule, such as substitution by half the detection limit, censored-data modeling or multiple imputation, depending on the assay and missingness structure. Variables measured on different analytical platforms should not be pooled directly until batch effects and platform-specific scaling have been assessed.

Missing data should be explicitly characterized rather than ignored. Missingness should be classified, where possible, as missing completely at random, missing at random or missing not at random. Missing not at random should be considered when the absence of a measurement may iftself reflect a biological or clinical state, such as failure to collect a sample because of diarrhea, antibiotic exposure, disease severity or low biomass.

Variables with very high missingness should be excluded from the primary NTI model or reserved for exploratory analyses. Moderate missingness can be addressed using multiple imputation, k-nearest-neighbor imputation or model-based imputation, with the method selected according to the variable type, distribution and biological plausibility. Importantly, imputation must be performed within the training set during model development to avoid information leakage into the validation set. The proportion of missing values for each proxy, the imputation strategy and complete-case sensitivity analyses should be reported.

Outliers should be evaluated in a structured manner. Extreme values may reflect true biological states, such as severe inflammation, very high bile-acid concentrations or highly disrupted microbiota after antibiotics, but they may also reflect technical artefacts. Therefore, outliers should first be assessed using sample metadata, assay-quality metrics, replicate consistency and batch information. Technical outliers should be excluded using predefined criteria. Biologically plausible extreme values may be retained, winsorized or robust-scaled, but the chosen approach should be tested in sensitivity analyses. Robust scaling using the median and interquartile range may be preferable when distributions remain non-normal after transformation.

Continuous variables should be transformed when appropriate to reduce skewness. Highly skewed proxies, including metabolite concentrations, cytokines and taxon abundances, can be log-transformed before scaling. Standardization can then be performed using Z-scoring:

$$z_{i}=\frac{x_{i}-\mu }{\sigma },$$

where $x_{i}$ is the observed proxy value, μ is the cohort mean and σ is the standard deviation. This transformation places variables with different units on a comparable scale and prevents numerically large variables from dominating the index. Binary variables can be encoded as 0/1, ordinal variables can be rank-transformed or treated as ordered factors, and bounded variables can be min–max scaled when their interpretation depends on a fixed biological range.^67^

If the NTI proxy set includes compositional microbiome variables, such as relative abundances derived from 16S rRNA sequencing, shotgun metagenomics or metatranscriptomics, these variables should not be analyzed directly on the relative-abundance scale. Because sequencing data are constrained by a fixed library size or total-sum constraint, an apparent increase in one taxon necessarily induces apparent decreases in others, even when their absolute abundances have not changed. This closure effect can generate spurious correlations and distort downstream estimates of niche occupancy, functional redundancy and resource overlap.^64^ Therefore, compositional features should be transformed before inclusion in the NTI workflow.

A commonly used approach is centered log-ratio (CLR) transformation:

$$\mathrm{CLR}\left(x_{j}\right)=\;\ln\left(\frac{x_{j}}{g\left(x\right)}\right),$$

where,

$$g\left(x\right)={\left(\prod_{j=1}^{k}x_{j}\right)}^{1/k}$$

is the geometric mean of all (*k*) components in sample (*x*).^68^ Here, *x*_*j*_ denotes the abundance of component *j*, such as a taxon, gene family or metabolic pathway; *k* is the total number of measured components. The CLR transformation expresses each component relative to the geometric mean of the full composition, thereby reducing distortions caused by the unit-sum constraint. Because logarithms cannot be computed for zero values, zeros should be handled using a predefined pseudocount or zero-replacement method before transformation. The same zero-handling rule should be applied consistently across all samples, and sensitivity analyses should test whether different pseudocounts or zero-replacement strategies materially change the NTI estimate. This is particularly important for rare taxa, low-biomass samples and sparse metagenomic features.

After preprocessing, all proxies should be directionally aligned and placed on a non-negative scale before aggregation. Each exclusionary-force proxy should be oriented so that higher values indicate stronger resistance to engraftment, whereas each facilitative-force proxy should be oriented so that higher values indicate greater niche permissiveness. For example, a high abundance of closely related competitors, high functional redundancy and high resource overlap should increase *E*, whereas niche vacancies, compatible substrate availability or cross-feeding potential should increase *F*. Directionality should be specified a priori to avoid post hoc weighting decisions that artificially improve model performance. Because Z-scored variables can take negative values, standardized proxies should be converted into non-negative oriented scores before they are combined into *E* and *F*. This can be done using min–max scaling, percentile-rank transformation or another predefined monotonic transformation. The resulting oriented scores can be denoted as $s_{m}^{E}$ and $s_{n}^{F}$ , where higher values always indicate stronger exclusionary or facilitative force, respectively. The composite exclusionary score *E* and facilitative score *F* can then be calculated as weighted sums of these non-negative oriented scores:

$$E=\sum_{m=1}^{M}w_{m}^{E}s_{m}^{E},$$

$$F=\sum_{n=1}^{N}w_{n}^{F}s_{n}^{F}.$$

where $s_{m}^{E}$ and $s_{n}^{F}$ are non-negative oriented exclusionary and facilitative scores, respectively, and $w_{m}^{E}$ and $w_{n}^{F}$ are their corresponding weights. Weights should be non-negative and normalized within each proxy class so that $\sum_{m=1}^{M}w_{m}^{E}=1$ and $\sum_{n=1}^{N}w_{n}^{F}=1$ . Equal weighting can be used as a transparent baseline model, but it should not be assumed to be biologically optimal because different ecological barriers may dominate in different strain–host contexts. For example, resource overlap may be the main barrier for a nutrient-specialist bacterium, whereas bile-acid filtering, oxygen exposure or epithelial inflammation may dominate for strict anaerobes.

Weighting can be performed using three complementary strategies. First, expert-informed weighting can be applied when strong mechanistic prior knowledge exists, for example, when the metabolic requirements of the introduced strain are known. Second, unsupervised data-driven weighting can be used when the aim is dimensionality reduction rather than prediction. Principal component analysis or factor analysis can summarize correlated proxies into orthogonal components, although this approach may reduce biological interpretability. Third, supervised weighting can be used when engraftment outcomes are available. Logistic regression, random forests, gradient-boosting models or other supervised learners can identify proxies associated with strain persistence or functional engraftment. However, feature importance from non-linear models should be interpreted as predictive rather than causal and should not be treated as a direct biological weight.

Penalized regression provides a useful framework when the number of candidate proxies is large, proxies are correlated or the number of engraftment events is limited. For binary engraftment outcomes, penalized logistic regression minimizes the negative log-likelihood plus a penalty term. LASSO applies an L1 penalty^69^:

$$\min_{\beta }\left\{-l\left(\beta \right)+\lambda \sum_{j=1}^{p}\left|\beta_{j}\right|\right\},$$

whereas ridge regression applies an L2 penalty:

$$\min_{\beta }\left\{-l\left(\beta \right)+\lambda \sum_{j=1}^{p}\beta_{j}^{2}\right\}.$$

here, l(β) is the model log-likelihood, $\beta_{j}$ are proxy coefficients and λ controls the strength of shrinkage. LASSO can shrink some coefficients exactly to zero, thereby performing variable selection, whereas ridge regression stabilizes correlated coefficients without eliminating variables. Elastic net combines both penalties:

$$\min_{\beta }\left\{-l\left(\beta \right)+\lambda \left[\rho \sum_{j=1}^{p}\left|\beta_{j}\right|+\frac{1-\rho }{2}\sum_{j=1}^{p}\beta_{j}^{2}\right]\right\},$$

where 0≤ρ≤1 controls the balance between LASSO-like sparsity and ridge-like stabilization. Elastic net may be particularly appropriate for NTI modeling because ecological proxies such as α-diversity, resident-guild occupancy, functional redundancy and resource overlap are likely to be correlated.^70^ ^,^ ^71^

Once E and F have been calculated as non-negative composite scores, the NTI can be defined as a normalized contrast between exclusionary and facilitative forces:

$$\mathrm{NTI}=\frac{E-F}{E+F+\varepsilon },$$

where ε is a small positive constant that prevents numerical instability when *E + F* is close to zero. Under this convention, positive NTI values indicate a resistant niche in which exclusionary forces dominate, values near zero indicate a balanced or contested niche, and negative values indicate a permissive niche in which facilitative forces dominate. The value of ε should be fixed before analysis and assessed in sensitivity analyses because different choices may affect NTI estimates when both *E* and *F* are small. Alternative NTI formulations may be appropriate in specific biological contexts, but the directionality of the index must be fixed a priori and validated against observed engraftment outcomes.

Technical harmonization is required when NTI proxies are measured across different sequencing runs, centers, cohorts, extraction kits, metabolomics platforms or analytical batches. Batch effects should be assessed using negative controls, mock communities, replicate samples, batch-colored ordination plots and estimates of batch-associated variance. Empirical Bayes approaches such as ComBat can correct batch-specific shifts in mean and variance while preserving biological covariates, but they should be applied only after confirming that biological groups are not completely confounded with batch.^72^ In simpler settings, batch-specific centering and scaling may be sufficient. Quantile normalization can be considered when global distributions are expected to be comparable across batches, but it should be used cautiously because forcing all samples to share the same marginal distribution can remove genuine biological differences.^73^ When datasets cannot be harmonized at the raw-data level, random-effects meta-analysis can be used to combine proxy–engraftment associations across cohorts.

Collinearity among NTI proxies should be assessed before model fitting because many candidate variables may capture overlapping ecological information. For example, high α-diversity may be correlated with high functional redundancy, high resident-guild occupancy and reduced niche vacancy. The variance inflation factor (VIF) can be calculated for each proxy:

$${\mathrm{VIF}}_{i}=\frac{1}{1-R_{i}^{2}},$$

where $R_{i}^{2}$ is obtained by regressing proxy i on all other candidate proxies. VIF values substantially above 5–10 are commonly used as warning thresholds, although such cut-offs should be interpreted in context rather than applied mechanically.^74^ In high-dimensional settings, where the number of candidate proxies approaches or exceeds the number of participants, VIF estimates may themselves be unstable; in such cases, correlation clustering, dimensionality reduction or penalized modelling should be preferred. If problematic collinearity is detected, several remedies are available: principal component regression can replace correlated predictors with orthogonal components; ridge or elastic-net regression can stabilize correlated coefficients; variable clustering can group related proxies into latent ecological domains; and hierarchical modeling can assign weights at both the domain and within-domain levels. Where a causal structure is hypothesized, structural equation modeling may help distinguish direct effects, such as resource overlap, from indirect effects mediated by diet, inflammation or community diversity.

Once these sources of preprocessing, batch-related, and collinearity-driven distortion have been addressed, NTI can be evaluated as a predictive variable rather than only as a descriptive ecological score. The relationship between NTI and engraftment probability can then be modeled using logistic regression:

$$P(\mathrm{engraftment})=\frac{1}{1+\exp[-(\alpha +\beta \;\cdot \;\mathrm{NTI})]\;}$$

where α is the intercept and β estimates the change in engraftment probability per unit increase in NTI. If NTI is scaled so that higher values indicate stronger ecological resistance, β is expected to be negative, because a higher NTI should reduce the probability of engraftment. However, this relationship should be treated as a testable hypothesis, not as an assumed property of the index. In early applications, NTI should be evaluated both as a continuous predictor and as a stratification variable, for example, by comparing low-, intermediate-, and high-tension strata. This would determine whether NTI provides a monotonic predictor of engraftment probability or whether clinically useful thresholds are required.

Preliminary validation should use existing microbiome-intervention datasets in which baseline microbiome data and post-intervention strain-level engraftment are both available. Suitable datasets include controlled probiotic-colonization studies with individualized stool or mucosal engraftment outcomes, post-antibiotic probiotic-recovery studies, FMT datasets with donor–recipient strain tracking, and live biotherapeutic trials with baseline metagenomics and longitudinal strain detection. Human probiotic-colonization studies are particularly relevant because they show that probiotic persistence can be highly individualized and associated with baseline host and microbiome features.^25^ Post-antibiotic recovery studies can test whether NTI captures transient permissive windows created by ecological perturbation.^75^ FMT strain-tracking datasets can be used to evaluate whether pre-treatment recipient ecology, donor strain abundance and donor–recipient phylogenetic relatedness predict engraftment.^54^ ^,^ ^76^ ^,^ ^77^

Validation should be performed at several levels. First, the model should test whether NTI predicts binary engraftment, defined as detectable persistence of the introduced strain above a predefined abundance or prevalence threshold. Second, NTI should be evaluated against quantitative engraftment, such as post-dosing strain abundance, duration of persistence or area under the strain‒abundance curve. Third, when available, NTI should be tested against functional engraftment, such as recovery of a strain-specific metabolic pathway, production of a target metabolite or modulation of a host biomarker. Finally, the model should determine whether NTI adds predictive value beyond simpler baseline features, such as α-diversity, baseline abundance of related taxa, recent antibiotic exposure, the stool pH or dietary fiber intake. Ideally, the NTI model should be trained on one dataset and externally tested on an independent dataset generated by a different center, platform or intervention type.

Model performance should be evaluated using both discrimination and calibration. Discrimination can be assessed using receiver operating characteristic curves and the area under the receiver operating characteristic curve (AUROC). When engraftment is uncommon or when the proportion of engrafters is highly imbalanced, precision–recall curves and the area under the precision–recall curve (AUPRC) may be more informative. Calibration should be assessed using observed-versus-predicted engraftment plots, calibration intercept, calibration slope and Brier score, because a model can rank participants correctly yet still overestimate or underestimate the absolute engraftment probability.^78^ Decision-curve analysis can be used to determine whether NTI-based stratification provides clinically useful net benefits compared with treating all participants, treating none, or using simpler eligibility criteria.^79^

If sufficiently harmonized external datasets are unavailable, simulation-based validation should be used as an interim strategy. Synthetic microbial communities can be generated with predefined levels of diversity, resident-guild occupancy, functional redundancy, resource overlap, cross-feeding potential, bile-acid filtering, pH constraints, antibiotic perturbation and batch noise. Engraftment outcomes can then be simulated under known parameter values, allowing investigators to test whether the NTI workflow recovers the expected relationship between ecological resistance and strain establishment. Simulation scenarios should vary sample size, engraftment prevalence, effect size, sequencing depth, missingness, zero inflation, proxy noise, batch effects, correlation among predictors and the strength of unmeasured confounding. Because the true parameter values are known in simulation, this approach can estimate the bias, variance, calibration error, overfitting, false-positive rate and robustness of the NTI model under controlled conditions.^80^

Simulation-based validation should also test failure modes. For example, simulations should examine whether NTI remains stable when one proxy class is systematically missing, when a batch is partially confounded with a treatment arm, when rare taxa dominate a CLR-transformed feature set, or when the true relationship between NTI and engraftment is non-linear. Non-linear effects can be evaluated using restricted cubic splines, generalized additive models or tree-based models, but such approaches should be externally validated before being used for trial stratification. If simulation results show that NTI is unstable under realistic missingness, sparsity or batch structures, the proxy set should be simplified before prospective clinical use.

Future NTI-based trials should include simulation-based power analysis rather than relying on generic sample-size rules. The required sample size will depend on engraftment prevalence, expected NTI effect size, the number of candidate proxies, proxy correlation, missingness, batch effects, anticipated model complexity and whether the endpoint is binary engraftment, quantitative strain abundance, functional engraftment or clinical response. For prediction-model development, sample-size planning should account for the number of candidate predictor parameters, the expected outcome fraction and the anticipated model performance, rather than relying only on simple events-per-variable rules.^81^ If the sample size is limited, the NTI proxy set should be reduced a priori, or dimensionality reduction and penalized modeling should be used to reduce overfitting.

Power simulations should estimate the number of participants needed to detect a prespecified association between NTI and engraftment probability with adequate power while controlling false discovery and maintaining acceptable calibration. For a binary engraftment endpoint, simulations should vary the expected odds ratio per unit increase in NTI, baseline engraftment probability, missing-data rate and number of candidate proxies. For quantitative strain abundance, simulations should vary the expected effect size, abundance dispersion, zero inflation and measurement error. For trial stratification, simulations should test whether NTI-defined low-tension and high-tension groups differ sufficiently in engraftment probability to justify enrichment, adaptive randomization, niche preconditioning or targeted dosing windows.

Finally, NTI models should undergo rigorous internal and external validation before they are used for prospective trial decisions. Internal validation can use bootstrapping, repeated cross-validation or training–holdout splits to estimate optimism and tune model parameters, such as the penalty parameter *λ* in penalized regression. External validation should test the final locked model in an independent cohort without reselecting proxies or refitting weights. At this stage, NTI should be regarded as a hypothesis-generating and trial-design framework rather than a clinically actionable biomarker. Before NTI is used to guide patient selection, dosing windows, niche preconditioning or clinical decision-making, its proxy set, weighting scheme, endpoint definition and analysis plan should be preregistered and validated across multiple independent cohorts, ideally spanning different centers, sequencing platforms, intervention types and host populations. Clinical use should require demonstration that a locked NTI model retains discrimination, calibration and decision-curve utility across these cohorts and adds predictive value beyond simpler baseline variables such as α-diversity, recent antibiotic exposure, baseline abundance of related taxa, stool pH or dietary fiber intake.^82^ Reporting should follow established prediction-model reporting principles, including clear description of candidate predictors, missing-data handling, model specification, internal validation, external validation and calibration.

## Physicochemical filters and nutrition

### Transit time and pH

**Figure 4.**
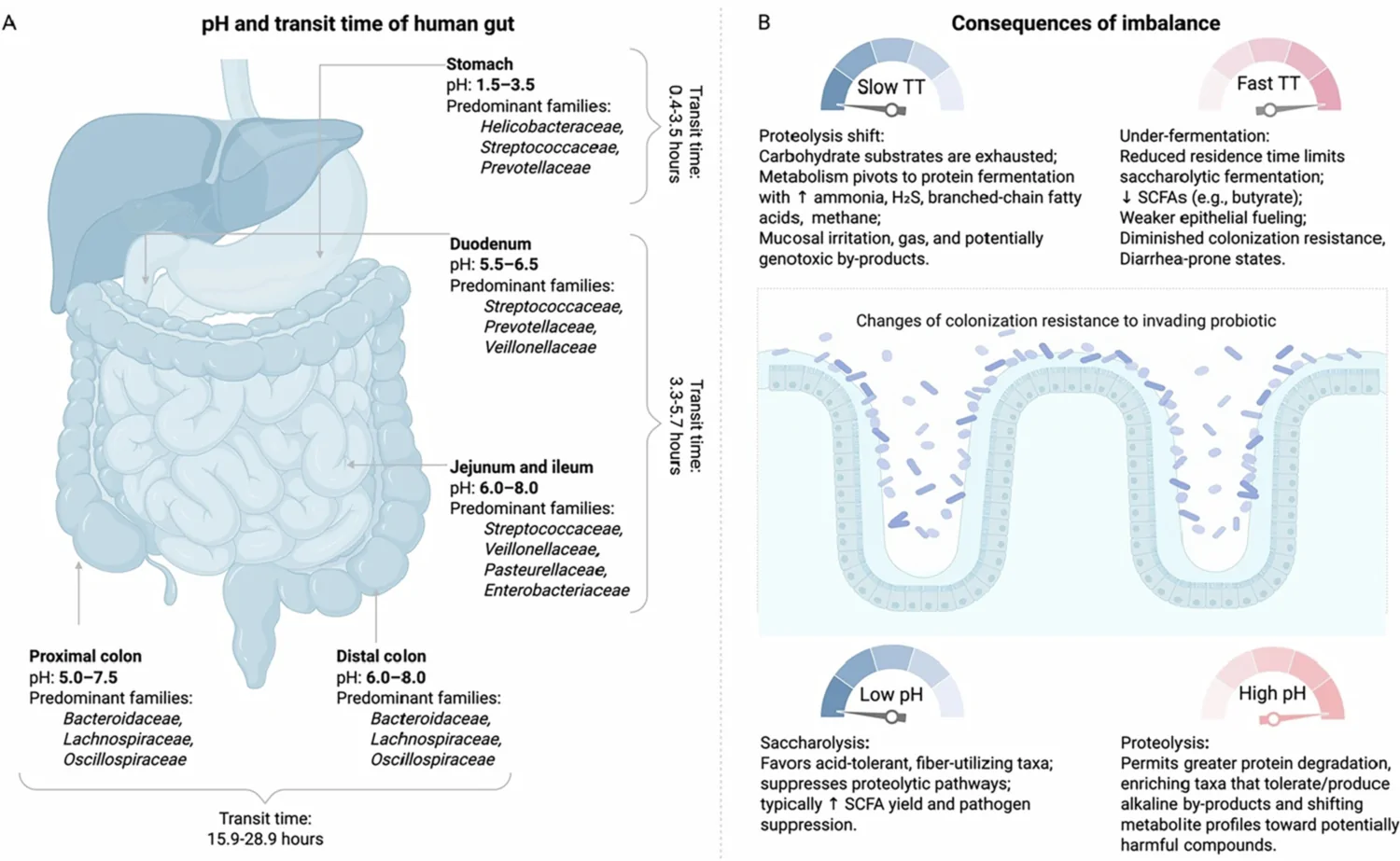
Transit time and pH as first-order ecological filters of the human gut microbiome and consequences of imbalance (a) pH and transit time of the human gut. Schematic map of regional physicochemistry and dominant taxa. (b) Consequences of imbalance. Slow transit shifts metabolism toward proteolysis with increasing ammonia, H₂S, branched-chain fatty acids and methane, causing gas, mucosal irritation, and potentially genotoxic by-products; fast transit limits fermentation, and decreasing SCFAs (e.g., butyrate), weakens epithelial fueling and colonization resistance, and predisposes to diarrhea. Low colonic pH favors saccharolytic fermentation, higher SCFA yield, and pathogen suppression; high colonic pH permits greater protein degradation and enriches taxa producing potentially harmful proteolytic metabolites.

Gut transit time and pH of gut vary markedly between people and are now recognized as a one driver of interpersonal differences in GM composition (Figure 4A).^83^ Even among healthy individuals on similar diets, those with slower intestinal transit tend to harbor distinct microbial communities compared to those with rapid transit.^83^ Longer transit allows microbes prolonged access to dietary residues, often shifting metabolism towards protein fermentation with increased production of proteolytic end-products (e.g., ammonia, hydrogen sulfide, branched-chain fatty acids) and gases like methane.^83^ In contrast, faster transit can limit the extent of fermentation.^83^ These effects are bidirectional as well—microbial metabolites can influence gut motility, creating feedback between the microbiota and transit dynamics.^83^

Regional pH gradients along the gastrointestinal tract impose an additional first-order selection on microbes. The stomach's low pH excludes all but acid-tolerant organisms, whereas the colonic pH is higher (typically within the 5–7 range) and shaped by microbial activity.^83^ Fermentation of carbohydrates yields organic acids that lower the colonic pH, which in turn inhibits pH-sensitive bacteria and favors acidophilic species.^83^ Fecal pH tends to be stable within an individual but varies widely between individuals, and these person-specific pH differences likely contribute to differences in their microbiome composition and metabolic profiles.^84^ Also, the colonic pH regulates which metabolic processes dominate: a more acidic colonic environment encourages saccharolytic fermentation and suppresses proteolytic pathways, whereas a higher pH can permit increased protein degradation by the microbiota.^84^ Thus, the gut's pH landscape—both the longitudinal gradient from the small to the large intestine and the absolute pH level unique to each host—acts as a key environmental filter for microbial colonists (Figure 4B).^84^

### Oxygenation and epithelial hypoxia as colonization switches

**Figure 5.**
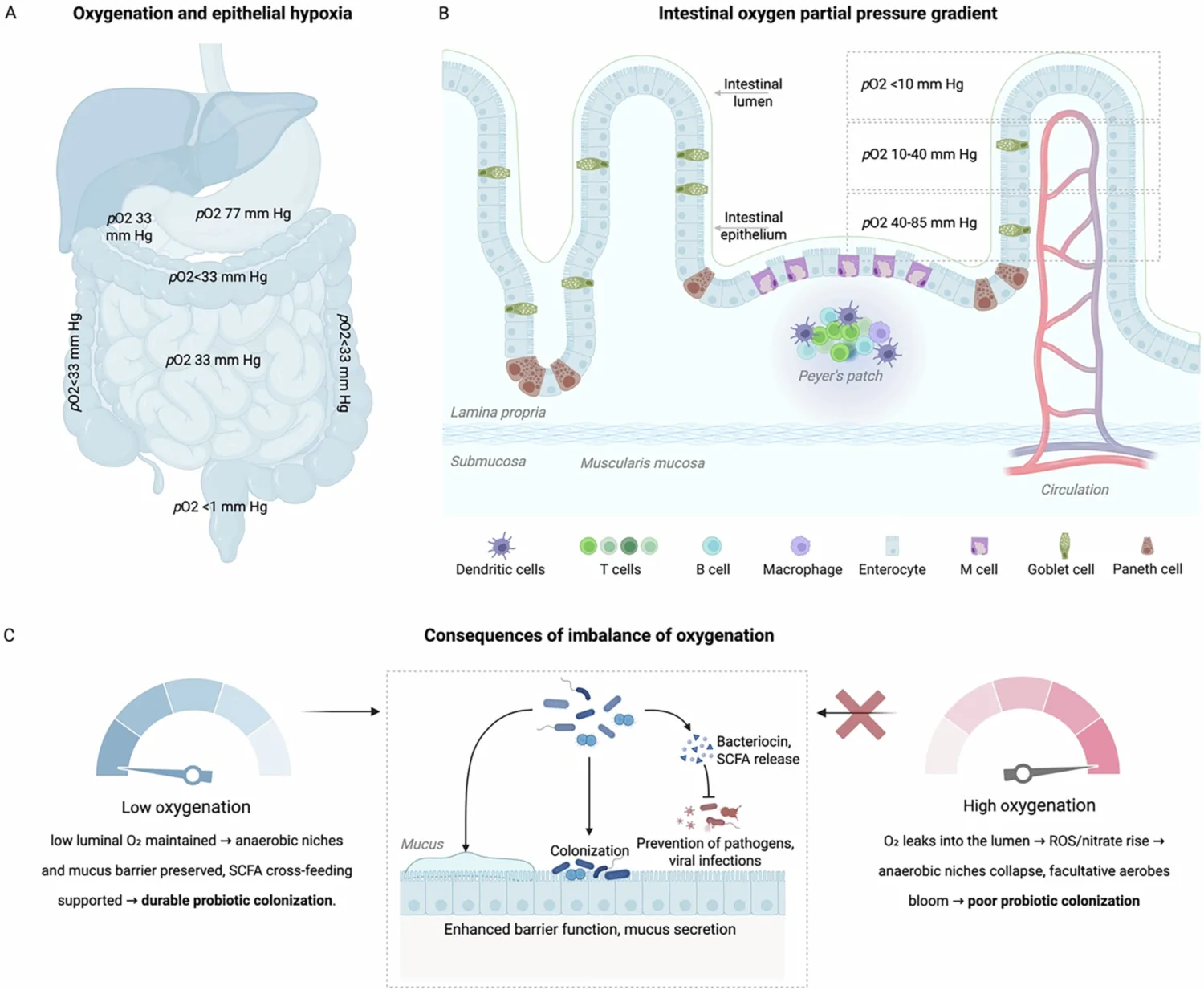
Intestinal oxygen gradients and their consequences for probiotic colonization. (a) Oxygenation and epithelial hypoxia (organ level). Map of the GI tract showing typical pO₂ values: higher near the proximal gut (≈77 mm Hg), ≈33 mm Hg across much of the intestine, and extremely low in the lumen/feces (<1 mm Hg), illustrating that intestinal epithelia exist in a relatively hypoxic environment. (b) Intestinal oxygen partial-pressure gradient (tissue level). Along the crypt–villus axis, oxygen falls from the epithelium to the lumen. Countercurrent capillary flow helps maintain low O₂ at the villus tip.^86^ (c) Consequences of imbalance of oxygenation (functional outcome) of low oxygenation and high oxygenation.

Oxygen availability in the gut is a pivotal environmental switch that can alter the microbial community structure and influence colonization resistance. In the healthy colon, the epithelium is in a state of physiological hypoxia (Figure 5A and B) — a condition actively maintained by both host and microbial processes. Commensal anaerobes (notably the butyrate-producing *Clostridia*) stimulate colonic epithelial cells to consume oxygen via mechanisms such as PPAR-γ activation, thereby limiting oxygen diffusion into the lumen.^85^ This epithelial hypoxia creates an anaerobic milieu in which obligate anaerobes flourish, and it suppresses the growth of facultative aerobes or fungi that would otherwise use oxygen to their advantage. In essence, limited oxygen acts as a barrier against intruders — if the gut environment remains highly reduced, many opportunistic microbes are kept in check by the resident anaerobic community (a key aspect of colonization resistance).

Conversely, a breach in this hypoxic barrier—for instance, owing to antibiotic therapy or inflammation—can switch the ecosystem into a state permissive for colonization by oxygen-reliant organisms.^85^ However, restoring epithelial hypoxia could prove sufficient to prevent this bloom. For example, treating mice with a PPAR-γ agonist (substituting for the missing butyrate signal) re-established a low-oxygen environment and thereby preserved colonization resistance, for example, against *Candida albicans* (*C. albicans*)^85^ which normally persists at low abundance as an anaerobiosis-limited minority. However, when oxygen became available post-antibiotics, the fungus could metabolize residual sugars and expand aggressively.^85^ Likewise, an oxygen-scavenging probiotic *E. coli* strain was only able to suppress *C. albicans* expansion when it was capable. Thus, shifts in gut oxygenation can act as a toggle for microbial colonization—when the epithelium remains hypoxic, it favors an anaerobic, colonization-resistant state, but if oxygen penetrates into the lumen, it tips the balance toward blooms of facultative pathogens and can impede the engraftment of strict-anaerobe probiotics (Figure 5C). Oxygen dynamics at the mucosal interface thus emerge as a sensitive “colonization switch” regulating which microbes dominate the gut ecosystem.^87^

### Nutrient blocking and diversity as community-level defenses

A diverse resident microbiota offers colonization resistance in large part by monopolizing nutrient niches, thereby starving potential invaders of the resources needed to establish. For example, systematic testing of gut bacteria in different combinations against the pathogens *Klebsiella pneumoniae* and *Salmonella enterica* showed that no single strain could reliably suppress pathogen growth, whereas multi-species consortia comprising dozens of members curtailed pathogen expansion by ~10^3^–10^4^ fold.^23^ The consortium composition was critical: assemblies containing key nutrient competitors achieved robust exclusion of invaders, while even species-rich communities lacking those competitors were comparatively permissive. Metabolic profiling (e.g., carbon-source utilization) further indicated that the strongest colonization resistance arose when resident communities substantially overlapped the pathogen's nutrient niche, thereby pre-empting resources required for its establishment.^23^In essence, a diverse community acts as a living “nutrient sink”, occupying a broad range of metabolic niches so that an invader finds few or no resources left unused. Under these conditions, an incoming microbe-even a pathogen-struggles to gain a foothold because incumbent bacteria have preemptively exhausted critical growth substrates.^23^ Conversely, maintaining a rich diversity-effectively filling the gut's metabolic “space”—is a community-level defense strategy that resists incursions. Not only the composition but also the external nutrient supply can tip this balance. For instance, it was shown that providing excess dietary amino acids can undermine colonization resistance: supplementing mice with certain amino acids abolished the microbiota's suppression of *Salmonella*, whereas restricting those nutrients (via a protein-free diet) dramatically enhanced resistance to *Salmonella* infection after antibiotic treatment.^88^ Such findings highlight that incumbent community diversity and nutrient occupation work hand-in-hand to exclude new microbes—either by direct competition for specific nutrients or by creating a resource-depleted environment hostile to would-be colonizers.^88^ In sum, a diverse, metabolically versatile microbiota defends its turf by leaving little nutritive opportunity for invaders, thereby serving as an ecological barricade against microbial colonization.

These results highlight that high microbiome diversity itself tends to correlate with protection, but more importantly, community composition and functional or metabolic capacity are decisive factors.

## Human-ecological modulators

### Sex-biased persistence of human gut strains

The host sex can exert a significant influence on which gut microbial strains persist over time. A large longitudinal meta-analysis of the fecal microbiomes from healthy individuals (12,415 metagenomes) revealed that certain bacteria—notably maternally inherited *Bifidobacterium* strains—tend to remain resident in females far more than in males.^27^ For example, dominant *B. bifidum* strains acquired in early infancy persisted beyond weaning in about 67% of girls compared with only 34% of boys, indicating greater strain-level stability in female infants' guts.^27^ This sex-biased persistence may reflect an ancient coevolution whereby beneficial maternal microbes are selectively retained in daughters (potentially aiding vertical transmission to the next generation).^27^

Even outside infancy, subtle yet consistent sex differences in GM composition have been documented. In a recent adult cohort, men showed slightly higher alpha diversity (Shannon index) than women and a distinct overall community profile (β-diversity), despite similar species richness. Machine-learning models could modestly predict sex from one's gut microbiome based on taxa abundance patterns; For instance, *Prevotella copri* tended to be more abundant in men, whereas genera such as *Blautia*, *Alistipes*, and others were enriched in women.^89^

Not only bacteria but also other microbial kingdom members exhibit sex-associated differences in colonization and persistence. Fungi provide a clear example: women have been shown to harbor a higher number and diversity of gut fungal isolates compared to men. ^90^ For example, females' stool samples grew more fungal species (on culture) and clustered separately from males', possibly because of hormonal influences on fungal growth or incidental transfer of vaginal yeasts such as *Candida* into the gut environment.^90^ On the other hand, certain pathogens and opportunists show male biases. In sum, an array of evidence now supports that sex is an important ecological modulator of the gut: influencing which microbes thrive or fade, from beneficial commensal strains to opportunistic invaders.

### Host-based persistence of human gut strains

Sex hormones and immune differences are key mechanistic factors shaping gender microbiome disparities. Hormone–microbe crosstalk has a bidirectional nature: on the one side, sex steroid hormones (such as estrogens, progesterone, and androgens) directly influence gut microbial growth and metabolism; on the other hand, gut microbes modulate circulating hormone levels (for example, via enterohepatic metabolism of steroids).^91^ Estrogen is a potent immune modulator that affects microbiota composition. Females' higher estrogen levels upregulate gut mucosal defenses; For instance, estradiol can increase mucus production and IgA, nurturing mucin-loving commensals such as bifidobacteria.^27^ Estrogen signaling skews immune responses toward enhanced tolerance and anti-inflammatory profiles in the gut: during *H. pylori* infection, estradiol elevates IL-10 and T-regulatory responses while dampening pro-inflammatory cytokines, thereby reducing tissue damage and the bacterial load.^92^ These hormone-driven effects help explain why women typically clear certain infections more efficiently than men. Indeed, adult females mount stronger innate and adaptive immune responses than males, leading to faster pathogen clearance (but also higher autoimmunity risk).^93^ Males, conversely, have higher androgen (testosterone) levels, which can be immunosuppressive and alter the gut ecology via metabolic routes. It has been demonstrated *in vivo* that testosterone skews the gut environment by modulating bile acids, thereby favoring a different microbial community structure.^94^ Removal of gonadal hormones or adding testosterone reversed or induced those microbiota shifts, confirming hormones as drivers of sex-specific microbiome composition.^94^ In short, intrinsic physiological differences—hormonal milieu and immune reactivity—create sex-specific selective pressures in the gut, allowing certain microbes to outcompete or persist better in one sex over the other.

Medication usage patterns and exposures that differ between men and women also contribute to microbiome differences. For example, women of reproductive age commonly use oral contraceptives (estrogen/progestin) and are more frequently prescribed antibiotics for recurrent UTIs, whereas men may have higher usage of other drug types; these habits impact the gut flora. A global analysis of gut resistomes found that in high-income countries, women carry ~9% higher antibiotic resistance gene load in their GM than men.^95^ This gap emerged in adulthood and is likely attributable to sex-specific antibiotic exposure and healthcare behaviors.^95^ Women's higher rates of antibiotic usage (e.g., for UTI prophylaxis or obstetric procedures) and healthcare contact can enrich antibiotic-resistant gut commensals, whereas men's lower healthcare engagement might leave them with fewer resistance genes.^95^ Beyond antibiotics, dietary and supplement differences along gender lines shape the gut ecosystem. Culturally, men tend to consume more protein and fat, and women more fiber and artificial sweeteners, which select for different microbial guilds. Women are also often encouraged to take iron and calcium supplements (for anemia or bone health), and these can substantially perturb the gut microbial balance. Oral iron supplementation, for instance, has been shown to “reprofile” the gut microbiome toward a dysbiotic state: iron increases luminal free iron that fosters the growth of enteropathogens (like *Escherichia* and *Salmonella*) at the expense of beneficial fermenters.^96^ Trials in infants and children report that adding iron drops or micronutrient powders leads to reductions in commensal *Bifidobacterium/Lactobacillus* and increases in potential pathogens (e.g., *Clostridium*) along with more gut inflammation.^96^ Thus, a seemingly simple sex-based difference (higher iron deficiency anemia in females, hence more iron supplementation) can create a cascade of microbial changes and “niche openings” for opportunists. Likewise, distinct intake of vitamins and amino acids may modulate sex differences in microbiota metabolism. For example, some evidence suggests that male-biased gut communities produce or degrade certain amino acids differently-in type 1 diabetes mouse model, and male microbiomes showed more impaired amino acid metabolism during disease development.^97^ Differential protein diets and muscle mass between sexes could alter available substrates (e.g., branched-chain amino acids or tryptophan) and their bacterial catabolites, potentially impacting metabolic and mood pathways uniquely in men and women.

Host genetic and disease factors tied to sex also intersect with microbiome ecology. Sex chromosomes themselves encode immune-related genes such as Toll-like receptor 7, IRAK1 on X-chromosome that can affect how the host recognizes and tolerates microbes.^93^ Females may thus respond with heightened inflammation to microbial components, contributing to their predisposition for autoimmune conditions, many of which involve dysbiosis. For example, diseases such as systemic lupus erythematosus or rheumatoid arthritis (predominantly female) have been linked to distinct GM signatures and aberrant immune–microbe interactions, partly owing to X-linked immune regulation and estrogen effects. On the other hand, certain hereditary or developmental conditions more common in males can shape the microbiome: a striking case is autism spectrum disorder, which has ~4:1 male prevalence and often features GI and microbiome alterations. Recent research posits that sex-specific gut–brain-axis factors, including microbial metabolites, may contribute to these neurodevelopmental differences.^91^ Additionally, sex differences in infectious disease susceptibility (beyond the gut) can feed back into the gut ecology. Men's higher propensity for severe infections (and interventions such as frequent hospitalizations) can lead to more exposure to opportunistic pathogens or broad-spectrum antibiotics, altering their baseline microbiota. Meanwhile, women's stronger vaccine responses and lower pathogen carriage rates for some infections mean their microbiomes might experience less pathogen-driven disruption.^93^ Even cancers show sex bias tied to microbiota: for instance, colorectal cancer incidence and response to immunotherapy differ between men and women, and the gut microbes implicated in modulating chemotherapy or checkpoint inhibitor efficacy vary by sex.^93^ Finally, parasitic organisms and other residents of the gut ecosystem must be considered.^98‐100^ Males and females often mount different immune responses to parasites -females tend to develop more robust Th2 immunity against helminths, for example, reducing worm burden, whereas males may harbor higher parasite loads.^101^ Parasites and similarly, fungi like *Candida* or *Protozoa*create “niche tension” with bacteria in the gut; sex-based immune pressures could tilt this competitive balance. In women, a faster clearance of certain parasites or yeast might free up niches that bacteria fill, whereas in men a heavier parasite colonization might suppress some bacterial competitors.

Lifestyle and psychosocial stress differences between genders offer yet another mechanistic layer. Chronic stress is known to disturb the gut microbiome via neuroendocrine and immune pathways, and these stress effects are sex specific. Experiments in mice have shown that under prolonged stress, female GM shifts and inflammatory markers occur sooner or differently than in males.^102^ Stress hormones such as cortisol can disrupt the intestinal barrier and microbial balance, but the magnitude and compensatory microbial changes depend on sex-linked HPA axis reactivity.^102^ For instance, it was found that transferring the GM from a stressed male to a female mouse could partially transfer male-typical immune responses, highlighting that microbial communities carry sex-specific immune signatures.^102^ Men and women also differ on average in diet quality, reaction on stress, sleep, exercise, and exposure to drugs, hormone profiles, all of which can potentially modulate the gut ecology. These influences collectively create different habitats in male and female guts, with varying resource availability and immune pressure shaping the microbial inhabitants.

### Probiotic engraftment in women and men

New clinical data suggest that the likelihood and duration of probiotic engraftment may differ between women and men. For example, in FMT used to treat refractory *C. difficile* infections, a recent analysis hinted that sex matching between donor and recipient improves engraftment outcomes. In a trial of FMT after stem-cell transplant, patients who received microbiota from a same-sex donor tended to achieve higher donor-strain engraftment in their gut than those with sex-mismatched donors.^103^

Similarly, it has been found that sex-discordant FMT (e.g., female patient receiving a male donor's stool) was associated with an increased risk of post-infection irritable bowel syndrome, compared to sex-concordant transfers.^103^ One hypothesis is that a “mismatched” microbiome may not integrate as smoothly because the host's hormonal and immune environment being adapted to a different equilibrium of microbes. Native bacteria in the recipient (shaped by the biological sex) could resist invasion more strongly if the incoming community evolved under another sex's conditions, leading to lower durability or dysregulated fermentation patterns manifesting as IBS symptoms.^103^ While more data are needed, these findings underscore that donor selection for microbiota therapies might be optimized by considering sex as a factor of engraftment efficiency. Beyond FMT, probiotic and live biotherapeutic products may also exhibit sex-specific performance. Strains that colonize well in females might not take hold as effectively in male guts, and vice versa. The previously mentioned bifidobacterial strains that show persistence in women^27^ could be strong candidates for probiotics targeted at female populations (For instance, to promote maternal–infant transfer of beneficial microbes). In fact, the sex-related resilience of certain *Bifidobacteria* was confirmed when examining clinical trial data: women supplemented with a “persistent genotype” bifidobacterial strain were more likely to maintain that strain long-term compared to those given a non-persistent variant.^27^ This suggests that tailoring microbial therapies to leverage strains that naturally thrive in one sex may improve their durability. On the other side, for conditions such as hepatic encephalopathy or metabolic syndrome where FMT or probiotics are considered, male patients might require different donor profiles or pre-conditioning to achieve lasting engraftment. There is also interest in how sex differences in immune surveillance could be manipulated to aid engraftment: for example, transiently dampening a female host's vigorous mucosal immunity might help colonize a typically male-associated microbe if that microbe is therapeutically beneficial, or conversely providing a competitive niche advantage (like specific prebiotics) for female-favored microbes in male hosts. Also, recognizing sex-based niche tensions has implications for preventing microbiome disruptions. Clinicians might weigh sex when prescribing microbiome-impacting treatments. A case in point is antibiotic stewardship: since women tend to carry more antibiotic-resistant flora,^95^ a female patient might need a different probiotic adjunct or follow-up monitoring after antibiotics to ensure recovery of a healthy microbiome. In men, who might be more prone to certain pathobiont blooms (For instance, testosterone-driven shifts favoring bile-tolerant anaerobes),^94^ different strategies could be used to maintain balance, such as post-antibiotic bile acid binders or specific dietary modifications. Thus, integrating sex as a variable in microbiome interventions can inform more personalized approaches, from matching FMT donors by sex to designing sex-specific consortia of probiotic strains to timing interventions around hormonal cycles. These strategies remain hypothesis-generating and should be tested prospectively before being used to guide donor selection, probiotic matching or dosing decisions.

### Sex, gender, and hormonal state as orthogonal axes of gut niche tension

Sex, gender, and hormonal state should be treated as distinct but interacting dimensions in microbiome and engraftment studies. In biological terms, sex usually refers to genetic, gonadal or anatomical attributes, such as XX versus XY chromosomes or reproductive anatomy, whereas gender refers to sociocultural identity and socially patterned exposures, including cisgender, transgender, and non-binary identities.^91^ The hormonal state is the dynamic endocrine context that can crosscut both sex and gender, including puberty, the menstrual cycle phase, pregnancy, menopause, hormonal contraceptive use and gender-affirming hormone therapy (GAHT). These dimensions should not be collapsed into a single variable: sex chromosomes and gonads influence the baseline hormone milieu and immune responses; gender can shape behavior, diet, stress burden and healthcare access; and circulating or exogenous hormones can directly modulate microbial niches.^91^ The bidirectional nature of this axis is illustrated by evidence that sex steroids shape the gut microbial structure, whereas gut microbes can regulate the levels of bioactive sex steroids.^91^ Empirical data illustrate these effects. During puberty, surges in androgens and estrogens drive the maturation of the gut, skin, and oral microbiomes. Although large high-quality human studies remain limited, animal and small-cohort studies suggest that puberty creates sex-specific microbial trajectories, paralleling neurodevelopmental effects.^91^ In menopause, loss of ovarian estrogen causes characteristic shifts: postmenopausal women exhibit gut microbiota profiles more similar to men (less diversity differences) and a “menopause paradox” in the vagina *Lactobacillus* dominance decreases while diversity rises.^104^ In large cohorts, higher estradiol has been associated with richer gut microbiota, including higher abundance of *Alistipes*, *Collinsella,* and *Clostridia* in women.^104^ Concurrently, *Porphyromonas somerae,* and *Atopobium vaginae* were enriched in postmenopausal endometrial cancer cases, suggesting estrogen-driven dysbiosis.^104^ Hormonal contraceptives also impact microbiota, for example, young women on combined hormonal birth control have distinct gut β-diversity and reduced abundance of several SCFA-producing taxa compared to non-users.^105^ These shifts could mediate known metabolic effects of contraceptives. Also, GAHT has been associated with microbiome shifts toward profiles linked to the affirmed gender and treatment-defined hormonal state. For example, it has been shown that trans men on testosterone lose vaginal *Lactobacillus* and gain gut-like taxa (*Dialister*, *Prevotella*), approaching a postmenopausal-like state (and that *Dialister/Prevotella* declines with longer therapy).^106^ Similarly, it was demonstrated that 12 weeks of GAHT in transgender individuals induced gender-aligned gut shifts: overall diversity was stable, but *Parabacteroides goldsteinii* and *E. coli* changed in abundance consistent with confirmed gender, and fatty acid metabolism genes shifted.^29^ Given these multifaceted effects, each dimension should be explicitly encoded and modeled as a distinct variable**-**including sex assigned at birth, gender identity, developmental stage (for example, puberty, and menopause), and exogenous hormonal exposures (such as contraceptives and gender-affirming hormone therapy), with appropriate control for multiple testing and confounding.

Analytically, sex should not be assigned a single default role across all NTI studies. Instead, its role should be prespecified according to the biological question, cohort composition, hormonal metadata, sample size, and evidence for effect modification. This recommendation is consistent with sex- and gender-aware reporting frameworks, which emphasize that sex and gender should be explicitly defined, analyzed and reported rather than treated as interchangeable background descriptors.^107^ ^,^ ^108^ A practical decision rule is useful: sex can be treated as an adjustment covariate when it is expected to shift the baseline microbiome or immune state but not the NTI-engraftment slope; it should be used as a stratification factor when endocrine or sex-specific ecological mechanisms are plausible but subgroup sample size is insufficient for independent model fitting; and separate sex-specific or hormonal-state-specific NTI models should be considered only when there is reproducible evidence that NTI weights, calibration or engraftment slopes differ across groups.

In the simplest setting, sex assigned at birth should be included as a covariate in a pooled NTI model when both sexes are represented, when there is adequate overlap in age, diet, medication exposure and hormonal state, and when there is no strong prior or empirical evidence that the association between NTI and engraftment differs by sex. This pooled approach estimates the average association between NTI and engraftment while adjusting for baseline sex-associated differences in microbiome composition, immune tone and the endocrine milieu. However, sex should not be treated merely as a nuisance variable. Experimental and human data indicate that sex steroids and the gut microbiome can interact bidirectionally, including through hormone-dependent regulation of microbial ecology and immune phenotypes.^109‐111^ Therefore, NTI × sex and NTI × hormonal-state interactions should be tested explicitly when sex-dependent ecological mechanisms are biologically plausible.

Sex should be used as a stratification factor when sex-specific ecological mechanisms are biologically plausible or prespecified, but the available data are insufficient to fit fully separate models. This includes studies in which puberty, menstrual cycling, menopause, hormonal contraceptive use or gender-affirming hormone therapy are expected to shift niche structure, but sample size within each subgroup limits independent estimation of all NTI weights. In this setting, the same NTI definition can be applied across strata, but model performance, calibration and estimated NTI–engraftment slopes should be reported separately for each sex or hormonal-state stratum. Stratified reporting is particularly important when pooled calibration is acceptable, but subgroup calibration differs because a model may rank participants correctly overall while systematically overestimating or underestimating engraftment probability in one endocrine subgroup. This issue is relevant because menopausal status, circulating estrogens, hormonal contraceptive exposure and gender-affirming hormone therapy have all been associated with measurable shifts in gut or genital microbial communities.^29,105,112‐114^

Separate sex-specific or hormonal-state-specific NTI models should be considered only when there is strong evidence that the relevant exclusionary and facilitative forces, their weights or their relationship with engraftment differ across groups. Examples include studies comparing premenopausal and postmenopausal women, cohorts with substantial gender-affirming hormone therapy exposure, or trials in which the candidate probiotic is expected to interact with sex-steroid-dependent bile-acid, immune or mucosal pathways. In such cases, *E* and *F* can be constructed from the same prespecified proxy classes but with group-specific scaling, weighting or coefficients, provided that each subgroup has sufficient engraftment events for stable estimation. If subgroup sample sizes are small, hierarchical or partially pooled models are preferable to completely separate models because they allow sex- or hormone-specific deviations from a shared NTI structure while reducing overfitting.

A practical modeling sequence is therefore recommended. First, fit a pooled base model including NTI and key confounders. Second, add sex assigned at birth, gender identity and measured hormonal state as covariates. Third, test prespecified interaction terms, particularly NTI × sex, NTI × estradiol, NTI × testosterone, NTI × menopausal status, NTI × hormonal contraceptive use and NTI × gender-affirming hormone therapy. Fourth, if the interaction terms are large, biologically coherent and improve calibration or discrimination in internal validation, we report stratified performance or develop subgroup-specific NTI calibration. A representative repeated-measures logistic model is:

$$\mathrm{logit}\left\{P(\mathrm{engraftment}ᵢⱼ)\right\}=\alpha +\beta_{1}NTI_{ij}+\beta_{2}S_{i}+\beta_{3}H_{ij}+\beta_{4}G_{i}+\beta_{5}(NTI_{ij}\times S_{i})+\beta_{6}(NTI_{ij}\times H_{ij})+C_{ij}\gamma +u_{i},$$

where *Sᵢ* denotes sex assigned at birth, *Hᵢⱼ* denotes measured or treatment-defined hormonal state at time point *j*, *Gᵢ* denotes gender identity or gendered exposure category where relevant, *Cᵢⱼ* denotes confounders such as age, diet, body mass index (BMI), antibiotics and medication use, and *uᵢ* is a participant-level random intercept for repeated sampling. In this framework, a non-significant and poorly predictive interaction supports the use of sex as an adjustment covariate, whereas a reproducible NTI × sex or NTI × hormonal-state interaction supports stratified reporting or separate NTI calibration. Importantly, hormones should not be adjusted for indiscriminately. If the scientific question concerns the total effect of sex on microbiome niche tension, hormones may lie on the causal pathway and should be analyzed using mediation or structural models rather than simply controlled away. Conversely, if the goal is to estimate the direct association between NTI and engraftment independent of the endocrine state, measured hormone levels and exogenous hormonal exposures should be included as covariates or time-varying predictors.

Biological sex should therefore be operationalized according to cohort structure. In cohorts composed entirely of cisgender participants, sex assigned at birth can be coded as a binary or categorical variable, depending on the available metadata and the study question. In cohorts including intersex participants or other sex-development categories, sex should be represented as a categorical factor with explicitly reported levels rather than forced into a binary coding scheme. When the goal is descriptive inference, estimates should be reported by sex where sample size allows; when the goal is prediction, calibration and discrimination should be checked within sex-defined strata even if the final NTI model is pooled. This avoids a common failure mode in which a model performs adequately in the combined cohort but is systematically miscalibrated in one sex or endocrine subgroup.

Gender identity should be represented as a categorical variable or by dummy variables, depending on sample size and model structure. When microbiome composition or engraftment is expected to vary according to socially patterned or gendered exposures, such as healthcare utilization, dietary practices, stress burden, medication use or other behavioral determinants, gender identity should be included as a covariate or used as a stratification factor. In smaller cohorts, the effect of gender identity may be difficult to disentangle from sex assigned at birth and hormonal exposure, particularly in studies involving GAHT, where gender identity, sex assigned at birth and endocrine state are often strongly correlated. In such settings, propensity-score weighting, inverse-probability weighting or related balancing approaches should be considered to reduce confounding and improve comparability across groups.

Puberty and menopause should be treated as biologically meaningful transition states rather than crude age proxies. They can be encoded as binary indicators, ordinal developmental stages or continuous age-based variables modeled with non-linear terms. For example, puberty can be represented using Tanner stage, circulating sex-steroid concentrations or age-spline terms centered around the expected window of pubertal maturation. Menopausal status can be coded as premenopausal, perimenopausal or postmenopausal or as a binary variable when metadata are limited; spline regression with knots placed across the typical age range of perimenopause can capture gradual endocrine decline more faithfully than a simple linear age term. In studies of menstrual cycling, cycle phase and, ideally, directly measured hormone concentrations should be modeled as time-varying covariates in longitudinal analyses, for example, by representing estradiol as *E₂(t)*, thereby allowing temporal fluctuations in the endocrine state to be linked explicitly to within-individual microbiome dynamics.

Exogenous hormonal exposures, including hormonal contraceptives and GAHT, should be modeled as time-varying treatment variables rather than as static baseline characteristics. Depending on the available metadata, these exposures can be represented using binary indicators, such as current user versus non-user; categorical variables capturing formulation, route or regimen; and continuous measures, such as the cumulative dose, treatment duration or time since initiation. In longitudinal settings, these variables should be incorporated into mixed-effects models either as fixed effects or as explicitly time-varying covariates. Where treatment history changes over time and is itself influenced by prior covariates, causal approaches such as marginal structural models with inverse-probability weighting may be more appropriate. Interaction terms with sex, age, menopausal status, baseline microbiome configuration or NTI should also be considered, as they may reveal effect modification that would be obscured in pooled analyses. Because the initiation and continuation of hormonal therapy are rarely random, propensity-score methods or inverse-probability weighting are recommended to reduce confounding by indication and improve comparability between exposed and unexposed groups.

Rigorous multiplicity correction, such as Benjamini‒Hochberg false-discovery-rate control, should be applied when testing multiple hormonal, sex-related, microbial or interaction terms are tested. Key confounders should be modeled explicitly, including diet, BMI or adiposity, recent antibiotic exposure, medication use and lifestyle variables. Core models should incorporate sex–hormone axes rather than sex alone, for example, by including estradiol, progesterone, testosterone, hormonal birth-control exposure or GAHT, where these variables are relevant and available. Non-linear effects, such as splines for hormone concentrations, interaction terms and mixed-effects structures for repeated measures or batch effects, should be prespecified where biologically justified. When hormones plausibly lie on the causal pathway between sex and microbiome composition or function, formal causal mediation analyses or structural equation frameworks should be used to decompose total, direct and indirect effects rather than adjusting hormones away indiscriminately.

Prespecified sensitivity and stratified analyses should be conducted to check robustness. Relevant examples include excluding participants with recent hormonal contraceptive use, analyzing premenopausal and postmenopausal women separately, repeating models with and without directly measured hormone concentrations, and testing alternative cut-offs for GAHT duration, such as less than 6 months versus at least 6 months, with additional continuous-duration models where possible. Falsification or negative-control analyses, such as permuted gender labels, biologically implausible exposures or outcomes not expected to be hormone-sensitive, can help estimate the background rate of spurious associations. Data-adaptive components should be tuned transparently: spline complexity and knot placement can be optimized using information criteria or penalization approaches, and propensity-score models should be evaluated using cross-validation, balance diagnostics and overlap checks rather than discrimination alone. Wherever feasible, key associations should be validated in independent external cohorts, and the analytical framework should be preregistered to minimize model shopping and selective reporting. Taken together, careful operationalization of sex, gender and endocrine exposures, combined with appropriately specified longitudinal and causal models, should improve the ability of future studies to distinguish biological hormonal effects from socially patterned exposures in shaping microbiome niche tension and engraftment.^29^ ^,^ ^91^

### Microbe-host and microbe‒microbe interactions in the spatial domain

Recent studies reveal that the gut microbiota's spatial organization-from the mucus layer to the crypts and lumen is a key determinant of microbe–host and microbe–microbe interactions, shaping what we term niche tension. High-resolution spatial transcriptomics and imaging (e.g., μm-scale spatial RNA-seq) now map microbial taxa and host gene expression across the gut landscape.^115^ ^,^ ^116^ These data show distinct mucus‐associated communities (e.g., anaerobic, Clostridia-rich biofilms) separated from lumenal bacteria,^117^ with strain‐level preferences driven by adhesion genes.^116^ Gradients of oxygen, pH, and secreted host factors (e.g., mucus glycoproteins, antimicrobial peptides) interact with microbial motility and chemotaxis: for example, modeling and microfluidic gut‐mimetic experiments demonstrate that flow‐driven AI-2 gradients and chemotaxis cause *E. coli* to accumulate in crypt-like niches and form biomass “hotspots” that self-limit via nutrient/oxygen depletion.^118^ Concurrently, gut bacteria appear to use positional cues from host-secreted molecules to regulate costly functions: the spatial sensing hypothesis predicts microbes suppress or upregulate public-goods production depending on epithelial proximity.^119^ At the microscale, interspecies interactions (competition, cross-feeding, antagonism, quorum sensing) further impose niche tensions. Agent-based metabolic models show that oxygen and nutrient gradients create spatial partitioning of metabolism and cross-feeding between species.^120^ *In vitro* co-cultures with mucin hydrogels reproduce mucosa & lumen stratification, revealing that particular strains and genes (e.g., adhesion factors) govern mucosal colonization, while others prefer the lumen.^116^ Host responses add another layer: spatial transcriptomics has uncovered pockets of epithelial immune activation (e.g., interferon-λ and innate sensor signatures) precisely co-located with clusters of luminal bacteria.^121^

Together, these findings illustrate how the 3D gut architecture, chemical gradients, and dynamic behaviors generate a complex tension of niches: microbial communities are simultaneously constrained by host barriers and immune microenvironments, while microbes exploit or reshape local conditions (via motility, aggregation, or metabolism) to establish advantageous niches. Quantitative modeling and emerging spatial ecological metrics (e.g., from individual-based models) are beginning to formalize this “niche tension,” demonstrating how spatial organization governs microbiome composition and function. These concepts, mapping key host and environmental factors with their mechanistic roles and levels of evidence for influence on community structure and probiotic establishment, are presented in Table 2.

**Table 2.** Evidence summary of determinants of microbial ecology and probiotic colonization.

Modifier	Community structure evidence	Engraftment/clinical evidence	Evidence level
pH gradients^84^	Strong synthesis that GI pH varies regionally; fecal pH stable within-person but varies across persons; colonic pH regulates fermentation pathways and metabolic output	Indirect: pH as “filter for colonists” is plausible but requires probiotic-engraftment trials stratified by measured segmental pH	Indirect/hypothesis-generating
Transit time/motility^83^	Mentioned as driver of community differences; direct mechanistic/quantitative transit → engraftment predictability not established here	No direct probiotic timed-by-transit evidence	Indirect/hypothesis-generating
Oxygen gradients/epithelial hypoxia^85^	Mechanistically links oxygen microenvironment to ecological state (hypoxia supports anaerobe-dominated colonization resistance)	Direct: antibiotic-induced oxygenation enables *Candida* bloom; restoring hypoxia or adding oxygen-respiring probiotic suppresses bloom	Mechanistic/experimental
Diet/nutrients/fiber^23^ ^,^ ^61^	Nutrient blocking and diversity-dependent resistance; diet and metabolites as ecological constraints	Direct clinical moderation shown via baseline niche occupancy (Akkermansia supplementation depends on baseline abundance), and diet-holding conditions included in some RCTs	Direct clinical moderation/partly indirect
Bile acids^122^	Present as plausible chemical stressors and signaling molecules; detailed bile-acid→probiotic outcome experiments not retrieved	No direct predictable probiotic-response evidence	Indirect/hypothesis-generating
Antibiotics/concomitant drugs^85^	Antibiotics as a major perturbation and driver of ecological state changes	Direct: antibiotics create permissive state for *Candida*; probiotic oxygen respiration reduces bloom; pharmacologic restoration of hypoxia restores resistance	Mechanistic/experimental
Host immunity/inflammation^24^	Immunologic context is presented as a filter; colonization resistance mechanisms overview	Direct engraftment → clinical predictability not retrieved; evidence mainly conceptual/review-level	Indirect/conceptual
Mucosal niche occupancy/spatial structure^115^	Spatial transcriptomics enables mapping of host–microbiome interface across sites and micron scales; supports “spatial niche” as real and measurable	No direct probiotic outcome stratified by mucus & lumen occupancy	Indirect/hypothesis-generating
Small intestine & colon differences^84^	Demonstrated in pH gradients and spatial mapping across intestinal regions (proximal SI, ileum, caecum, colon).	No direct “delivery to SI & colon” outcome predictability	Indirect/hypothesis-generating
Sex/sex hormones^91^ ^,^ ^97^ ^,^ ^102^ ^,^ ^123^ ^,^ ^124^	Sex-biased microbial persistence strategies across life (human microbiome persistence genetics)	Direct probiotic engraftment differences by sex	Emerging/partly direct
Propagule pressure/dose/delivery^125^	Dose appears as an invasion-ecology determinant; direct dose–response predictability varies by strain/ecosystem and needs trial data	Direct dosing regimens exist in probiotic RCTs (e.g., 10^10^/day dosing frameworks), but cross-dose causal inference is limited without multi-dose arms	Indirect/trial-design rationale
Timing (circadian/feeding)^12^	Microbiota–circadian coupling is demonstrated; diet timing shapes oscillations conceptually	Chrono-probiotics remains a hypothesis; there is not direct clinical trials testing dosing time as a determinant of engraftment	Hypothesis-generating

## Design rules for precision probiotics

Several translational concepts discussed below, including sex-aware probiotic selection, chrono-probiotics, AI-assisted engraftment prediction and personalized niche preconditioning, should be interpreted as hypothesis-generating strategies unless supported by prospective, controlled and externally validated human data.

### Niche pre-conditioning via diet and microenvironmental factors

Successful probiotic engraftment begins with pre-conditioning the host niche—tailoring the gut environment through diet, timing, and other factors.

Dietary fibers are a prime example because fermentable fibers can shape microbial metabolism toward beneficial pathways. For instance, adding fermentable fibers was shown to suppress the production of harmful indole metabolites and instead promote the generation of beneficial tryptophan metabolites via cross-feeding among gut bacteria.^126^ Also, there are synthesized evidence that circadian clocks and the gut microbiota are bidirectionally coupled.^127^ ^,^ ^128^ Feeding timing and composition (e.g., high-fat & fiber-rich diets) shape microbial diurnal oscillations; in turn, microbial metabolites (notably SCFAs, bile acids, and tryptophan/indole derivatives) act back on host clocks, influencing lipid metabolism and energy homeostasis.^122^

Probiotic supplementation may influence host rhythmic physiology, supporting the concept of chrono-probiotics, in which strain- or metabolite-targeted probiotics are aligned with circadian and feeding cycles. In principle, timed dosing could help stabilize peripheral gut clocks, reduce circadian disruption caused by jet lag or shift work, and create more favorable windows for strain engraftment or metabolic activity. However, this remains a hypothesis-generating strategy rather than an established clinical intervention. Direct evidence that probiotic dosing time improves strain engraftment, persistence or therapeutic response is currently lacking, and any benefit is likely to be strain-specific and dependent on timing relative to feeding, light exposure and host sleep–wake rhythms. Future studies should therefore test chrono-probiotic strategies using prespecified time-of-dosing arms, longitudinal strain tracking and metabolomic readouts.

Controlling other aspects like gut oxygen levels can also be critical, especially since many next-generation probiotics are strict anaerobes.^129^ ^,^ ^130^ One solution is to engineer oxygen tolerance or pair strains with oxygen-consuming partners-as shown for *Faecalibacterium prausnitzii*, which was co-isolated with a sulfate reducer and then evolved for oxygen resilience, producing a symbiotic formulation that colonized some human participants.^129^

Beyond diet and oxygen, host physiological factors play a major role in niche readiness. Hormonal and immune milieus can profoundly influence probiotic activity. There is a bidirectional hormone–microbe crosstalk in the gut: commensal bacteria not only respond to host hormones but can also produce hormone-like signals, affecting the host in turn.^131^

The host immune status is a further consideration: probiotics tend to engraft better in an immune-compatible environment, whereas inflammation may hinder them or skew their function. Medication usage patterns—especially antibiotics—are perhaps the most immediate niche disruptors. Broad-spectrum antibiotics can decimate commensals and any co-administered probiotic strains, undermining probiotic benefits during treatment.^132^ Indeed, patients commonly take probiotics to restore microbiome balance during or after antibiotics, yet these antibiotics often eliminate probiotic bacteria before they can act.^132^ To overcome this, innovative strategies such as protective coatings have emerged. For example, a “nanoarmor” coating for probiotic cells (made of edible tannic acid–iron complexes) has been shown to protect both Gram-positive and Gram-negative probiotic strains from multiple antibiotics.^132^ Such armored probiotics survived antibiotic co-treatment, successfully colonizing the gut and preventing antibiotic-associated dysbiosis and diarrhea.^132^ This demonstrates how niche pre-conditioning can be extended to shielding probiotics from hostile factors (such as drugs or bile) until they safely reach their target niche.

Thus, precision probiotic design must account for the myriad factors shaping the gut ecosystem before and during the introduction of a microbe. Diet (fibers, micronutrients, and even amino acid supplements) can be tuned to favor the probiotic's growth and metabolite profile.^126^ Environmental conditions (pH, oxygen, and moisture) can be modulated, or microbes can be bioengineered to tolerate them.^129^ Hormonal or immune perturbations (due to stress, illness, or genetics) should be mitigated since a hormone-balanced and immunologically “receptive” gut is more amenable to colonization.^12^ Even co-existing microbiota and pathogens matter: the presence of certain pathogens or prior infections might demand pre-emptive measures (e.g., antiparasitic treatment or an anti-inflammatory diet) to clear space for the probiotic. By pre-conditioning the niche through diet, timing, and management of host factors, clinicians can substantially improve the engraftment and efficacy of precision probiotics.

### Resource overlap and co-operative guilds

Another design rule for precision probiotics is ecological matching, ensuring that the introduced microbes fit the resident microbial community and resource landscape. Rather than adding a single strain and hoping that it overcomes all competitors, researchers now recognize the importance of *avoiding direct resource overlap* and even fostering cooperation. In natural ecosystems, multiple species coexist by partitioning niches and resources, preventing any one species from a “winner-takes-all” domination.^133^ Imitating this, spatial or metabolic segregation can stabilize multi-strain probiotics. For example, encapsulated different bacterial strains (*E. coli*, *Saccharomyces cerevisiae*) in tiny polymer microcapsules, creating microbial swarmbots that physically keep strains slightly apart.^133^ Small molecules can diffuse between capsules, so bacteria still cross-talk and share metabolites, but spatial separation prevents them from directly out-competing each other for the same nutrients.^133^ These engineered consortium approach achieved a stable community where each strain maintained its population.

Beyond physical strategies, metabolic matching of probiotic strains is key. Precision probiotics increasingly involve consortia of microbes selected or engineered to work together as a guild rather than as single-strain supplements. These consortia are designed with complementary functions—one strain might produce a metabolite that another consumes, creating a productive loop instead of competition.^134^ A striking example is engineered a *Bacillus subtilis* strain to produce butyrate (a beneficial short-chain fatty acid) and used microfluidic screen to find a partner bacterium that could thrive on the metabolites that *Bacillus* produced.^134^

Metabolic profiling identified *Bifidobacterium pseudocatenulatum* as a cross-feeder on *Bacillus*-derived peptides and B6 vitamers; *in vivo*, the two-strain consortium outperformed a single strain in reducing obesity-associated endpoints in mice.^134^ The cooperative pair not only directly impacted host weight gain but also enriched other beneficial bacteria (such as *B. longum*) in the gut and modulated vitamin B6 metabolism in ways that a single strain could not.^134^ Another example is the co-culture of *F. prausnitzii* with its hydrogen-consuming partner *Desulfovibrio piger* (*D. piger*). *F. prausnitzii* produces butyrate but also lactate and hydrogen which accumulation feeds back negatively on fermentation. By pairing it with *D. piger*, which uses hydrogen and produces sulfate, created a mutualistic mini consortium that enhanced butyrate output and growth of the probiotic strain.^129^ These examples highlight how engineered guilds can yield synergistic effects: one microbe's “waste” becomes another's fuel, and together, they achieve a therapeutic outcome that is robust and sustainable.

Precision probiotics are designed by considering the existing microbiome ecology of the target individual and selecting strains (or communities of strains) that will integrate effectively. If a patient's gut is missing a function (e.g., fiber degradation or mucus utilization), a probiotic providing that function might fill the vacant niche. Conversely, if a niche is already crowded (e.g., multiple strains eating the same fiber), a new probiotic might need a different food source or a partner that modifies the environment in its favor. Modern synthetic biology offers tools to engineer such consortia, but even without genetic engineering, selecting naturally synergistic strains (from human microbiome databases or stool banks) is a viable strategy. By minimizing unproductive competition and maximizing cooperative interactions, ecological matching greatly improves the chances that a precision probiotic will engraft and deliver its benefits *in vivo.* ^134^

### Personalized and robust evaluation designs

Designing precision probiotics also calls for rethinking how we test and validate them in clinical trials. Traditional randomized controlled trials often average out the very individual differences that precision approaches seek to exploit. In response, researchers are proposing innovative trial architectures to better capture *who* benefits from a given probiotic and *under what conditions*. One such approach is N-of-1 crossover trials,^135^ ^,^ ^136^ where each participant alternates between the probiotic and a placebo (or alternate intervention) in multiple cycles. Rather than comparing large groups, an N-of-1 trial treats the individual as their own control, allowing the detection of personal responses. This could be especially useful in microbiome interventions, where heterogeneity is high—one person's “miracle probiotic” may do nothing in another. By having each subject go through periods on and off the probiotic, clinicians can identify responders more reliably (e.g., if a person consistently reports improved symptoms only during the probiotic periods, that is strong evidence of efficacy for that individual). Aggregating data from many N-of-1 trials can then reveal patterns (such as particular microbiome or genetic profiles associated with response) that a traditional trial might miss. While few published probiotic studies have fully adopted N-of-1 designs yet, the paradigm is gaining attention as a way to personalize evidence in the era of precision medicine.^135^ ^,^ ^136^ It aligns with recommendations to embrace more adaptive, individualized trial designs in microbiome research,^22,83,137‐140^ ensuring that beneficial effects are not “washed out” by non-responders in a large cohort.

Another recommendation for probiotic trial design is stratification by host factors such as sex and demographic characteristics. For example, analyses from the Human Microbiome Project and other studies have shown consistent sex-based differences in gut bacterial populations (e.g., certain *Bacteroides* & *Prevotella* abundances), and these differences become pronounced after puberty in tandem with sex hormone changes.^131^ It stands to reason that a probiotic might have different efficacy in males & females or might colonize differently due to these baseline distinctions. Sex-stratified randomization means ensuring that trials enroll balanced numbers of men and women (or males/females in animal studies) and pre-planning to analyze outcomes separately by sex. This approach can uncover if an intervention works better in one group or if dosing should differ. Similarly, other stratifications (by age, genetic risk factors, etc.) may be warranted in precision probiotic trials to account for sources of variability. The overarching principle is to design studies that do not treat the population as homogeneous but rather embrace variability—using it to learn for whom *and* under what conditions a probiotic is truly effective.

Finally, precision probiotics demand more nuanced endpoints and sampling strategies in trials. Traditional trials often rely on clinical endpoints (symptom scores, disease incidence) and coarse microbiome readouts (stool analysis before & amp; after treatment). However, stool sampling alone may miss the full story of probiotic colonization. The gut is spatially complex, and fecal bacteria represent mainly the distal colon contents -essentially a downstream effluent.^141^ Cutting-edge studies using ingestible sampling devices have revealed that microbial communities and metabolites in the small intestine or along the colon can differ significantly from those in stool.^141^ Key microbial activities (like bile acid transformation) were happening in specific gut regions and were largely complete by the time stool was formed.^141^ This means a probiotic could colonize or act in the upper gut or at the mucosal lining without large changes detectable in stool. Spatial sampling beyond stool, e.g., taking mucosal biopsies, colonic lumen samples via colonoscopy, or using swallowable devices that capture luminal contents can give a much clearer picture of where the probiotic is engrafting and functioning. Similarly, using engraftment-first endpoints has been proposed: before declaring a probiotic “effective” or not, first check if it took hold in the microbiome of each participant. To monitor how probiotics colonize the gut, GM sequencing must be performed to see if the probiotic strain increases in abundance or exhibits its functional signature (e.g., production of a particular metabolite). This is crucial because a lack of clinical effect might be due to a lack of engraftment rather than a truly ineffectual microbe. In one recent human trial of an oxygen-tolerant *F. prausnitzii* probiotic, researchers found the strain detectable in only a subset of treated individuals.^129^ These data underscore why documenting colonization is important—it helps identify “non-engrafters” who might need additional niche pre-conditioning or an adjusted strain. Going forward, precision probiotic trials are likely to include *microbiome endpoints* such as “Did the probiotic survive and integrate?” and *spatial endpoints* such as “Where in the gut did it colonize or act?”. These outcomes, measured perhaps after a short engraftment period, can then inform or even replace longer-term health endpoints in early-phase trials.

This phased and comprehensive trial architecture—incorporating individualized or N-of-1 designs, stratification by host factors, spatial sampling beyond stool and engraftment tracking—may help evaluate precision probiotics more rigorously. However, these approaches should be treated as trial-design strategies rather than evidence that personalized probiotic interventions are already clinically validated.^141^

## Data-driven prediction of colonization outcomes

At present, data-driven prediction of probiotic engraftment should be considered exploratory. Machine-learning, network-based and digital-twin approaches can generate useful hypotheses and identify candidate ecological predictors, but most available models have not yet undergone prospective, multi-cohort clinical validation. Their outputs should therefore be interpreted as decision-support hypotheses rather than as validated clinical recommendations.

Beyond rigorous reporting standards, researchers are turning to computational tools to predict probiotic engraftment in individuals—a step toward personalized microbiome therapeutics. Several emerging platforms combine host and microbiome data to forecast whether a probiotic will successfully colonize a given gut environment. Below, we compare three approaches that exemplify the state-of-the-art, noting their advantages, limitations, validation status, and treatment of host sex.

### Machine-learning classification models

Fully data-driven models have been applied to learn patterns in pre-intervention microbiomes that predict probiotic colonization outcomes.^26^ For example, Random Forest and neural network models have been trained on hundreds of baseline community profiles to classify permissive & “resistant” gut communities for a given probiotic species. A recent study achieved high accuracy in predicting engraftment of *E. faecium* and *A. muciniphila in vitro* using fecal consortia as training data.^26^ These models can leverage complex, high-dimensional microbiome features without requiring explicit mechanistic knowledge, successfully identifying subtle compositional signatures that favor engraftment.^26^ Their advantages are that they are often open source and can be implemented quickly once a labeled dataset becomes available. However*,* predictive performance depends on having large, well-annotated training sets, which in human trials can be a bottleneck. Models may overfit to specific study conditions or species and provide less insight into why a probiotic fails or succeeds (a “black box” issue). Notably, most current ML engraftment models have been validated only in controlled laboratory or synthetic community settings, not prospectively in human trials. The incorporation of host factors such as diet or genetics is minimal so far, and sex is typically not explicitly included in the feature set, meaning that any sex-specific effects might be overlooked unless the model is retrained or stratified by sex.

### Integrated prediction platforms & other tools

Some efforts combine statistical and mechanistic elements or focus on specific clinical contexts. For instance, responder profiling algorithms have been used in FMT and synbiotic studies to match donors to recipients or to forecast outcome based on community similarity. The *iMic* algorithm could predict FMT success in recurrent *C. difficile* by analyzing donor microbiome features alone,^142^ highlighting that certain community compositions universally facilitate engraftment. For probiotics, a 2025 platform employed a link-prediction AI to map probiotic strains to likely health outcomes—essentially a network approach that, while aimed at therapeutic effect, can prioritize strains with higher odds of surviving and functionally integrating *in vivo.* ^143^

Another emerging concept is that the digital twin of the gut is based on using individualized microbiome data to simulate how an ecosystem will react to an intervention. In an early demonstration, an AI-driven digital twin of infant guts successfully forecasted microbiome trajectory changes over time,^144^ which could eventually be extended to testing virtual probiotic “engraftment” scenarios for a given host. These integrative platforms may incorporate multi-omics data (microbial genetics, metabolites, and host gene expression) and can be tailored to specific use-cases (e.g., sex-specific predictions in women's health probiotics—enhancing mechanistic research on precision probiotic therapies (R61/R33 Clinical Trial Optional)). Their advantage is that they often produce interpretable rules, for example, identifying that a person lacking *B. infantis* and with low gut diversity is an ideal candidate for *B. infantis* probiotic engraftment. Many such tools are in prototype or preclinical stages. Proprietary platforms if developed by biotech companies might not be fully transparent. Moreover, the sex factor is just beginning to be recognized; few tools explicitly include sex as a variable yet. There is a push from funding agencies to address sex influences in precision probiotic research, and initial evidence of sex-biased microbial colonization^27^ suggests that next-generation prediction models will need to stratify or adjust for sex to improve their accuracy.

Thus, data-driven and hybrid models are rapidly advancing the ability to forecast probiotic colonization outcomes before a trial or therapy is deployed. While pure machine-learning models excel at pattern recognition and have shown high predictive performance in experimental settings, mechanistic models enrich understanding by pinpointing the ecological interactions (e.g., nutrient competition) that drive engraftment. A combination of these approaches may yield the best of both worlds—for example, using mechanistic insights to inform feature selection for ML or using AI to identify which host and microbiome variables (including sex) to feed into simulations. Crucially, as these platforms mature, their validation in human studies will be the next benchmark. Early signs are promising (e.g., a defined bacterial consortium VE303 guided by such modeling has shown efficacy in preventing *C. difficile* recurrence but broader clinical trials integrating predictive tools are needed. Moreover, explicitly accounting for sex as a covariate remains an important frontier: future tools must ensure that the “niche tension” dynamics for probiotics are optimized for both male and female physiology, aligning with the sex-aware perspective of the human gut ecosystem. Table 3 provides a summary of recommended reporting parameters for probiotic engraftment trials.

**Table 3.** Recommended reporting parameters for probiotic engraftment trials.

Parameter	Rationale
Probiotic strain metadata: strain identity, genetics, formulation (e.g., CFU dose, viability)	Different probiotic strains (even within one species) can have markedly different colonization abilities and host effects. Precise strain reporting prevents result inconsistency and allows replication. For example, contradictory outcomes in weight management trials have been attributed to varying *Lactobacillus* strains^143^
Baseline microbiome composition: Taxonomic and functional profile (pre-intervention)	The pre-existing gut community structure strongly influences whether an introduced probiotic will find an available niche or be excluded. High community richness and the presence/absence of specific taxa can predict engraftment success or failure. Trials should report baseline 16S rRNA/metagenomic profiles so that engraftment can be interpreted in the context of each host's initial microbiome
Microbiome sequencing methods: techniques used (16S rRNA gene, shotgun metagenomics, virome, culture) and resolution	The method of microbiome analysis determines the resolution of engraftment detection. Strain-level engraftment often requires shotgun metagenomic sequencing or strain-specific qPCR, whereas 16S rRNA may only confirm genus-level presence. Trials should report sequencing platforms, depth, and any pipelines for strain tracking. This transparency ensures that engraftment (e.g., of a probiotic strain & indigenous near-relatives) is assessed appropriately and that studies are comparable^27^
Host age and sex (and sex-specific endpoints): age and sex of participants and analysis stratified by sex	Host sex can modulate microbiome ecology and probiotic engraftment. Certain bacteria exhibit sex-biased persistence in humans, suggesting hormonal or immunological differences in niche favorability. Reporting outcomes by sex (or ensuring balanced recruitment) is crucial in a “sex-aware” probiotic ecology to detect differential efficacy or colonization patterns in males & females^27^
Dietary intake and fiber:baseline diet and diet during trial (especially fiber/prebiotic intake)	Diet shapes the gut's nutrient landscape and can enhance or hinder probiotic engraftment. High-fiber diets, For instance, cultivate microbiomes that are more receptive to fiber-fermenting probiotics. Conversely, high sugar or specific nutrient intake might interact with probiotics to alter outcomes. Documenting dietary patterns (or controlling diet) allows interpretation of engraftment in light of feeding the niche^137^ ​​​​​​
Patients' diseases—genetic, hormonal, metabolic, oncological, etc.)	Underlying disease states substantially reshape the gut ecosystem by altering host-derived selective pressures that govern microbial survival, colonization, and persistence. Genetic variants can influence mucosal immunity, barrier function, bile acid metabolism, and nutrient availability, thereby modifying colonization resistance^145^ Hormonal states, such as sex hormone levels, menopause, androgen deprivation, or endocrine disorders-affect immune tone, gut motility, and microbial metabolic networks, leading to sex- and hormone-dependent differences in engraftment capacity^27^ Metabolic diseases, including obesity and diabetes, are associated with chronic low-grade inflammation, altered bile acid pools, and shifts in microbial functional niches, which can either impede or facilitate probiotic establishment^146^ Oncological conditions and their treatments (e.g., chemotherapy, immunotherapy, radiation) induce profound ecological perturbations, including mucosal damage, immune dysregulation, and transient niche vacancies, resulting in highly variable engraftment outcomes^147^
Concomitant medications and exposures—especially recent antibiotics, prebiotics, or FMTs	Medications or interventions that perturb the gut ecosystem can drastically alter niche availability. Notably, antibiotic use prior to or during a trial lowers microbiome diversity and can promote colonization by exogenous species (including probiotics) by clearing competing flora. Other factors like prebiotic supplements or fecal microbiota transplantation also shift community structure. Recording these data ensures that engraftment (or lack thereof) can be attributed to the probiotic rather than confounders^26^

## Conclusions

The variable efficacy of probiotics and live biotherapeutic products in clinical trials is not merely a matter of selecting an inappropriate strain or administering an insufficient bacterial dose but rather a predictable consequence of attempting to introduce novel microorganisms into a highly structured and already occupied ecosystem. This review proposes that probiotic success or failure is largely determined by the ecological state of the intestine at the time of administration, an environment shaped by physicochemical filters (pH, transit time, oxygen gradients, bile acids, etc.), host-related constraints, and the competitive architecture of the resident microbiome. To render this framework clinically applicable, we expanded colonization resistance via introducing the concept of microbial niche tension. This term is defined as a trial-operational state variable reflecting the net balance between exclusionary forces (competition, antagonism, spatial crowding, and host defense mechanisms) and facilitating forces (niche vacancy, cross-feeding, and permissive temporal windows). Moreover, the proposed NTI may serve as an additional conceptual and quantitative bridge between invasion ecology and precision microbiome therapeutics, enabling a shift from generalized supplementation toward stratified, context-dependent interventions; however, this requires further investigation. The introduction to the definition of the NTI in correlation with engraftment is still theoretical, but it provides a “blueprint” that connects exclusionary forces (e.g., competition and host defence) and facilitative forces (e.g., niche vacancy and cross-feeding). We should highlight that engraftment, as a standalone linear parameter, correlates with clinical aftermath, but only when it is defined by spatially localized, functional niche occupancy. Stool testing is often a non-specific marker; while the NTI quantifies the engraftment probability, the clinical outcome is determined by other endogenous standard host factors. Following this sequence, engraftment is a prerequisite for an outcome but not a guarantee of clinical benefit. Recent FMT studies further support that clinical benefit tracks more closely with durable acquisition of specific beneficial strains and functions than with total engraftment alone.^43^ The introduction to the definition of the NTI in correlation with engraftment is still theoretical, but it provides a “blueprint” that connects exclusionary forces (e.g., competition and host defence) and facilitative forces (e.g., niche vacancy and cross-feeding). Therefore, future research should approach NTI as a hypothesis to be tested using independent, pre-registered, multicenter spatial datasets with longitudinal outcomes. Future studies should move forward from single-cohort effect sizes and seek out-of-sample validation and employ hierarchical spatial models. Confirmation of spatial microbiome claims requires multimodal, pre-registered, independent empirical datasets linked to clinical outcomes. This should be accompanied by a pre-specified analysis plan detailing the required sample sizes and power targets, which should be determined via simulation.

Importantly, niche tension is not uniform across individuals, intestinal compartments, or time. It varies with diet, antibiotic exposure, circadian rhythms, and spatial microenvironments, and is further modulated by host-specific factors, including sex and hormonal status. These variables remain insufficiently studied in most probiotic investigations, despite increasing evidence for sex-biased microbial persistence and engraftment dynamics. Recognition of these modulating determinants reframes non-response not as probiotic failure but as ecological incompatibility: many probiotics are simply unable to achieve stable engraftment or exert meaningful functional effects in high-tension niches where resources are pre-empted and host filters remain restrictive.

Collectively, the implications of this can be useful for the development of next-generation probiotics. Effective microbiome interventions will require niche preconditioning (dietary, physicochemical, or pharmacological), cooperation-based consortia rather than direct competitive strategies, and engraftment-prioritized clinical studies employing strain-level tracking and deeper sampling approaches that extend beyond stool specimens alone.

Finally, integrating mechanistic ecological reasoning with data-driven predictive tools, including machine learning models and emerging digital-twin approaches, may provide a future pathway to forecasting who is most likely to respond, provided that such models are externally validated, calibrated and tested prospectively.

Within this framework, probiotics can be conceptualized as candidate precision ecological therapies rather than universal supplements, but this framing requires prospective validation before clinical implementation. However, NTI should not be used as a clinical decision tool until its proxy structure and predictive performance have been preregistered and validated in multiple independent cohorts.
